# Diversity dynamics of microfossils from the Cretaceous to the Neogene show mixed responses to events

**DOI:** 10.1111/pala.12615

**Published:** 2022-07-15

**Authors:** Katie M. Jamson, Benjamin C. Moon, Andrew J. Fraass

**Affiliations:** ^1^ 8205 Palaeobiology Research Group School of Earth Sciences University of Bristol Wills Memorial Building, Queens Road Bristol BS8 1RJ UK; ^2^ The Academy of Natural Sciences of Drexel University 1900 Benjamin Franklin Parkway Philadelphia PA 19103 USA; ^3^ Present address: 8205 School of Earth & Ocean Sciences University of Victoria Bob Wright Centre A405 Victoria BC V8W 2Y2 Canada

**Keywords:** microfossil, diversity dynamics, macroevolution, PyRate, Bayesian, selectivity

## Abstract

Microfossils have a ubiquitous and well‐studied fossil record with temporally and spatially fluctuating diversity, but how this arises and how major events affect speciation and extinction is uncertain. We present one of the first applications of PyRate to a micropalaeontological global occurrence dataset, reconstructing diversification rates within a Bayesian framework from the Mesozoic to the Neogene in four microfossil groups: planktic foraminiferans, calcareous nannofossils, radiolarians and diatoms. Calcareous and siliceous groups demonstrate opposed but inconsistent responses in diversification. Radiolarian origination increases from *c*. 104 Ma, maintaining high rates into the Cenozoic. Calcareous microfossil diversification rates significantly declines across the Cretaceous–Palaeogene boundary, while rates in siliceous microfossil groups remain stable until the Paleocene–Eocene transition. Diversification rates in the Cenozoic are largely stable in calcareous groups, whereas the Palaeogene is a turbulent time for diatoms. Diversification fluctuations are driven by climate change and fluctuations in sea surface temperatures, leading to different responses in the groups generating calcareous or siliceous microfossils. Extinctions are apparently induced by changes in anoxia, acidification and stratification; speciation tends to be associated with upwelling, productivity and ocean circulation. These results invite further micropalaeontological quantitative analysis and study of the effects of major transitions in the fossil record. Despite extensive occurrence data, regional diversification events were not recovered; neither were some global events. These unexpected results show the need to consider multiple spatiotemporal levels of diversity and diversification analyses and imply that occurrence datasets of different clades may be more appropriate for testing some hypotheses than others.

Preserved remains of marine plankton and algae are highly abundant, cosmopolitan, and well represented in the geological record (Bé & Hutson 1977; Norris 2000). These marine micro‐organisms are inextricably linked to their environment and their species and genera richness fluctuates through time (e.g. O’Dogherty & Guex 2002; Erba 2004; Lazarus *et al*. 2014; Fraass *et al*. 2015). The exceptional preservation of microfossils provides a unique opportunity to investigate diversity dynamics, selectivity of extinction events, and sensitivity of certain ecological groups to different global‐scale environmental forcing events (Bown *et al*. 2004; Ezard *et al*. 2011; Lowery *et al*. 2020), including fluctuating atmospheric CO_2_ concentrations, sea surface temperatures (SSTs), and ocean circulation patterns. Additionally, biological interactions, such as symbiotic relationships, are catalysts for the evolutionary changes in major microfossil groups (e.g. photosymbiotic species (Norris 1996; Birch *et al*. 2012)). However, these must be assessed against impacts upon microfossil preservation, abundance, diversity and assemblages (Ezard *et al*. 2011; Pälike *et al*. 2012; Campbell *et al*. 2018). The improved temporal resolution of all major marine microfossil records has allowed investigation of the impacts of macroevolutionary events upon species‐level diversity (Lowery *et al*. 2020).

Numerous extinction events and major perturbations in climate have punctuated Earth’s geological history, varying spatially and temporally in intensity, tempo and selectivity, evidenced in the sedimentological record (Raup & Sepkoski 1982; Erwin 2001; Foote 2010). From the Cretaceous and throughout the Cenozoic, a myriad of different events occurred, each with different drivers and unique impacts upon and within different microfossil groups. The Cretaceous–Palaeogene boundary (K/Pg) is an example of one of the most significant and instantaneous extinctions triggered by a bolide impact on the Yucatán carbonate platform at *c*. 66 Ma (Alvarez *et al*. 1980; Schulte *et al*. 2010; Dinarès‐Turell *et al*. 2014). This global‐scale event instigated prolonged darkness, global cooling, and severe disruption to the ocean’s biological and chemical processes (Coxall *et al*. 2006) and subsequently impacted significantly on diversity and survivability at all trophic levels. Examples of large‐scale climate changes have also impacted significantly on microfossil diversity and assemblage composition, such as the global temperature increase of 5–8°C at the Paleocene–Eocene transition (*c*. 56 Ma) (McInerney & Wing 2011). The origination of the event is debated, yet common hypotheses suggest volcanism or extensive dissociation of methane hydrates triggering rapid short‐term warming, giving rise to the Paleocene–Eocene Thermal Maximum (PETM; Dickens *et al*. 1995; Petrizzo 2007; Andrade *et al*. 2018). This event subsequently affected microfossil diversity, dissolution and preservation, with many groups experiencing large shifts in geographical range, rapid evolution and changes in trophic ecology (Scheibner *et al*. 2005; Mutterlose *et al*. 2007; Jiang *et al*. 2018). Today, the current trajectories of contemporary climate change show that anthropogenic influence will continue to alter our complex marine ecosystems, posing an imminent threat of transitioning into the next global extinction (Ceballos *et al*. 2015; IPCC In Press). Rapidly increasing carbon dioxide levels and subsequent atmospheric temperature increase is just one of the ways that humanity is putting marine ecosystems under persistent stress (IPCC In Press). Despite current climate change having some unique characters relative to the geological record, the resulting successive biodiversity losses necessitate an investigation of the impact of past extinction events on microfossil diversity and assemblages to allow for predictions of how different marine micro‐organism groups may respond and recover today (Erwin 2001; McInerney & Wing 2011).

Microfossil diversity studies frequently restrict analyses to the first and last occurrences, and the ranges established by those first and last occurrences of taxa (Ezard *et al*. 2011; Fraass *et al*. 2015). These methods ignore key components that are integral to the cause and maintenance of diversity: the complex interactions of speciation and extinction (Silvestro *et al*. 2014*a*, 2019). Relying solely on the raw fossil occurrence data poses problems with incompleteness and underrepresentation of many lineages, inconsistent sampling between clades (Foote *et al*. 2008), and it is unlikely to represent the true lifespan of a species (Alroy 2010). Several microfossil studies have tried to improve upon these methods by using subsampling and classic rarefaction curves (Rabosky & Sorhannus 2009; Lloyd *et al*. 2012*a*, *b* *;* Renaudie & Lazarus 2013; Kocsis *et al*. 2014; Lazarus *et al*. 2014) to standardize samples and account for heterogeneity in sampling (Close *et al*. 2018). This, however, promotes misleading interpretations of occurrence data, compresses species richness ratios, and flattens diversity trajectories (Cárcer *et al*. 2011; Chao & Jost 2012). Recent methodological advances in phylogenetic methods provide detailed assessments of evolutionary dynamics through time in many organisms. Planktic Foraminifera have had their evolutionary relationships comprehensively examined throughout the Cenozoic (Aze *et al*. 2011; Ezard *et al*. 2011; Fordham *et al*. 2018), providing insights into diversification and ancestry. This method is often limited in power when estimating diversity as it is dependent on accurate phylogenetic tree estimation of evolutionary rates and times (Warnock *et al*. 2015; Stadler *et al*. 2018; Mitchell *et al*. 2019; Magee & Höhna 2021), which can be exacerbated when extant species are rare and extinction rates are high (Silvestro *et al*. 2014*a*, 2018, 2019). Extinct and extant data have been incorporated into stochastic birth–death models to improve estimations of diversity and diversification rates (Liow *et al*. 2010), however, few integrate sampling and fossilization processes within diversity estimates, promoting significant biases (Etienne & Apol 2009).

Microfossil groups have different preservation potentials, largely related to test composition. Preservation of planktic foraminiferans and calcareous nannofossils varies through time with the positioning of the carbonate compensation depth (CCD; Van Andel 1975; Pälike *et al*. 2012). The CCD is the depth at which carbonate delivered to the bottom of the ocean is matched by dissolution, as stability of calcium carbonate declines with increasing depth, resulting in sediments below this threshold devoid of carbonate (Ridgwell & Zeebe 2005; Greene *et al*. 2019). In contrast, radiolarians and diatoms build with silica, forming ornate skeletons and frustrules (Kling & Boltovskoy 1999). Biogenic silica formation depends on the balance between organic productivity and other controls such as dissolution, upwelling and accumulation rates (Egge & Aksnes 1992; Kidder & Tomescu 2016). Undersaturation of silica can initiate the dissolution of biogenic silica in the water column and diagenesis of siliceous marine sediments (Van Cappellen & Qiu 1997). Thus, radiolarians and diatom oozes have a reduced preservation potential compared to calcareous oozes and are often under sampled (D’Hondt 2005; Dutkiewicz *et al*. 2016).

Development of methods to quantify diversification rates has identified the need to reconstruct preservation rates as a key input. The recently developed software PyRate jointly determines lineage duration and diversification dynamics by teasing out speciation and extinction rates independently, accounting for fossilization and sampling processes from occurrence data, while integrating sources of uncertainty within the analysis to limit bias in results (Silvestro *et al*. 2014*b*, 2019). Using all possible data often neglected in other methods, including extant species (Silvestro *et al*. 2014*a*), PyRate implements a robust hierarchical Bayesian framework to determine the most appropriate birth–death model for the data to estimate diversification rate changes through time (Silvestro *et al*. 2018). Using comprehensive microfossil occurrence data from the Neptune database (Renaudie *et al*. 2020), we reconstruct changes in diversification rates through 140 myr of major transitions to provide insights into the primary drivers of ecological and evolutionary change within microfossils from the Mesozoic to the Pliocene.

## Material and method

### Data source

Foraminiferan data were retrieved on 20 February 2020, while other fossil group data were downloaded on 26 May 2020 from Neptune Sandbox Berlin (NSB), the latest development of the Neptune database (Renaudie *et al*. 2020). NSB is openly accessible but requires interested scientists to request a username and password. The dataset holds information of over one million species occurrences of several microfossil groups pulled from scientific ocean drilling expeditions (Spencer‐Cervato 1999): the Deep‐Sea Drilling Project, Ocean Drilling Program, the Integrated Ocean Drilling Program, and International Ocean Discovery Program. Four fossil groups were downloaded: planktic foraminiferans, calcareous nannofossils, diatoms and radiolarians using the package ‘NSB Companion’ v2.1 (Renaudie *et al*. 2020) within R v4.0.2 (R Core Team 2013). Downloaded species occurrence data spanned *c*. 150–0 Ma, containing corresponding sampling details and palaeogeographical information (see Data S1). Microfossil datasets used had different time ranges; occurrence records began at 150 Ma for calcareous nannofossils and radiolarians, planktic foraminiferan records commence from 120 Ma, and diatom records start from 60 Ma.

### Data manipulation

Using commands within ‘NSB Companion’, synonymous species were corrected in the dataset, removing non‐valid taxa. A dating uncertainty of ±0.25 myr was applied to occurrence sample ages, taken from standard deviations of NSB data (Spencer‐Cervato *et al*. 1994) and subsequent improved stratigraphy. The packages devtools v2.3.1 (Wickham *et al*. 2020), RCurl v1.98‐1.2 (Lang 2020), and packages within the tidyverse v1.3.0 (Wickham *et al*. 2019) were used to format the data into a table of taxon‐occurrence range entries. Utility functions provided alongside PyRate were used to extract single point ages for each occurrence sampled from a uniform distribution, bounded by the occurrence ranges that were input into the main PyRate program v3.0 (Silvestro *et al*. 2014*b*). This extraction procedure was replicated ten times to account for the range of uncertainty associated with each occurrence (see Data S2). Despite downloading data from 150–0 Ma, only the data between 140 and 5 Ma were analysed due to large uncertainty margins in both the earliest occurrences and in extant data. This uncertainty is associated with truncated maximum and minimum ages in the dataset, which produced erroneous results.

### Diversification modelling

PyRate estimates preservation rates using occurrences to infer speciation and extinction ages of individual taxa within a hierarchical Bayesian framework, from which rates of speciation and extinction through time were reconstructed (Silvestro *et al*. 2014*b*). Incorporating all fossil occurrences removes the artificial modifications associated with subsampling methods. PyRate implements three different preservation models (the default non‐homogeneous, homogeneous, and time‐dependent Poisson processes) that vary the allowed heterogeneity of the preservation rates through time (Table 1). A gamma‐distributed parameter was also incorporated into these models to account for heterogeneous preservation between species. Each of the four major groups was run in a separate analysis, while compositional effects on preservation rates were assessed by grouping calcareous microfossils (foraminiferans and nannofossils) and siliceous microfossils (radiolarians and diatoms) into two further analyses. PyRate analyses for each taxonomic group were run as array jobs of the ten replicates generated on the Blue Pebble cluster at the University of Bristol (Advanced Computing Research Centre; see Table 1) using the recommended reversible jump Markov Chain Monte Carlo algorithm, generating 10^7^ iterations, and sampling every 5000 iterations. Convergence was assessed in Tracer v1.7 (Rambaut *et al*. 2018) using the effective sample size (ESS) to ensure that the posterior distribution was suitably represented. Although not all model runs completed the iterations within the 72‐hour wall time, all model runs converged (ESS > 200) providing representative values for preservation rates and species longevity of each species.

**Table 1:** PyRate preservation models used to model speciation and extinction rates in all fossil groups, with the specifications and modifications made to find the best model for the data.

Model	Modifications
**NHPP: Non‐homogeneous Poisson process of preservation** Preservation rates change following a normal (bell‐shaped) distribution, for the duration of the lineage lifespan.	‐mG: to test the Gamma model of rate heterogeneity on all models. Accounts for preservation heterogeneity. The shape of the distribution is estimated from the data. Runs were completed with and without Gamma. After model testing, the model deemed the best would always be run with Gamma included.
	Sampling age errors to test for model sensitivity; all of the following ages (my) were run: ±0.25, ±0.20, ±0.15, ±0.10, ±0.05 myr.
	‐A 4: Reversible Jump Markov Chain Monte Carlo (RJMCMC): used on all other models as it is deemed the most accurate (Silvestro *et al*. 2014).
	‐edgeshift: when calculating rate shifts, the maximum and minimum boundaries of occurrences can cause apparent rate shifts in the data that reflect a potential sampling bias. We used ‐edgeShift to calculate the times of rate shift within fixed boundaries of 140–5 Ma to avoid erroneous results.
**HPP: Homogeneous Poisson process** Preservation rate is static throughout time and across lineages.	‐mG: see NHPP ‐edgeShift: see NHPP.
**TPP: Time‐variable Poisson process** A model that assumes preservation rates will change across certain time intervals but remain constant within timeframes; it tests whether preservation alters as a function of time rather than among lineages.	‐mG: see NHPP ‐edgeShift: see NHPP ‐pP: the preservation rate distribution shape and rate were changed from the default of ‐pP 1.5 1.5 to ‐pP 1.5 0; setting the rate to 0 allowed a rate parameter to be estimated from the data to reduce any subjectivity; the results showed no significant change to default and the default settings were used in subsequent TPP models. ‐qShift: the time intervals tested were changed; both epochs and ages of the geological record were run in the model. Using ages as pre‐defined timeframes produced the higher quality results with a reduced error margin and was used in all TPP tests on all microfossil groups.

Results of model selection are shown in Table 2.

### Model selection

Model selection for each microfossil group used a maximum‐likelihood framework to allocate the best fitting preservation. The fit of each model was calculated in the Akaike Information Criterion (AIC), providing a statistical method for assessing the fit of qualitatively different preservation models. Our main analyses used a standard precision of ±0.25 myr for each occurrence age, however, the precision of the Neptune data varies and is more precise than this. The sensitivity of the analyses to occurrence dating precision was investigated by running four additional PyRate analyses with decreasing occurrence ranges (±0.20, ±0.15, ±0.10, ±0.05 myr) using default preservation model settings (NHPP; Table 1). The results show no significant difference, suggesting dating uncertainty at this level does not impact upon the results (see Fig. S1).

The parameters estimated using PyRate that are presented in the Results section below are speciation rate, extinction rate and subsequent net diversification rates, as well as species longevity. These diversification rates are the number of speciation/extinction events per lineage per unit time (here per 1 myr, shown as ‘lineage^−1^Ma^−1^’; Silvestro *et al*. 2014*b*). For net diversification rates, positive values represent speciation whereas negative values represent extinction. Where appropriate we also include the 95% credibility interval (95% CI). Note that in the birth–death process, mean species longevity equals the inverse of the extinction rate. These diversification rates are calculated for the four individual microfossil groups, and calcareous and siliceous groups. The significance of rate changes is calculated using standard log‐Bayes factors (BF) thresholds that determine statistical support (Kass & Raftery 1995). A BF of 2 represents a positive signal while a BF of 6 represents a strong signal.

## Results

### Sampling and preservation

**Fig. 1:**
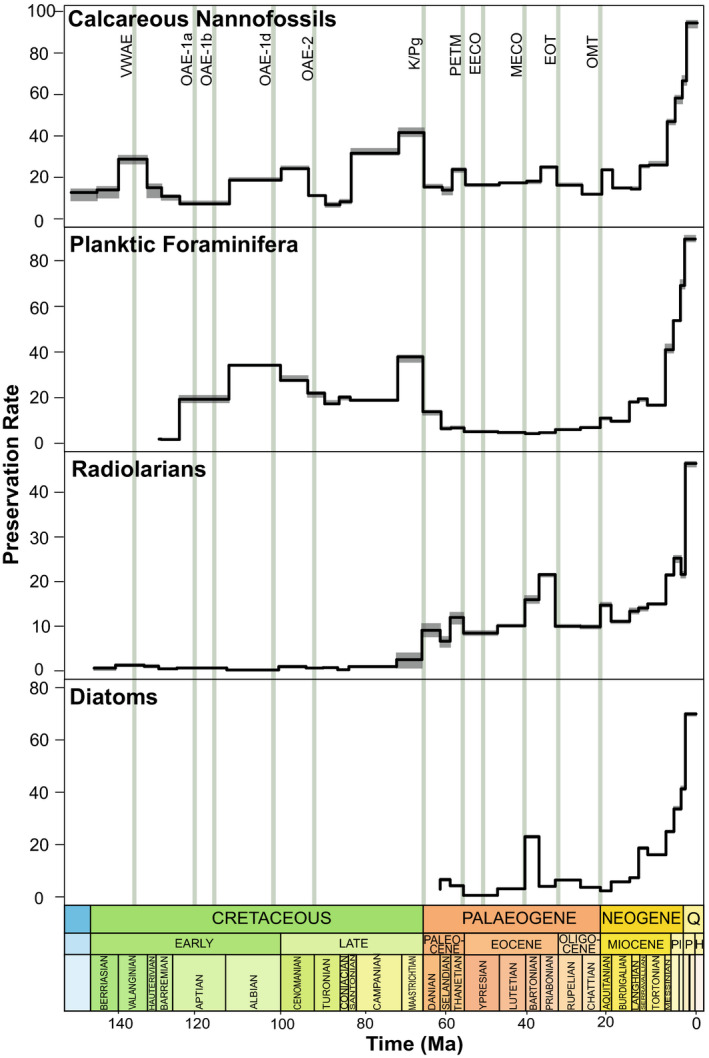
Preservation rates of each of the four microfossil groups from 150 Ma to the Neogene (occurrences lineage^−1^Ma^−1^). Preservation in all groups increases towards the Recent. Calcareous groups generally have higher preservation rates than siliceous groups. Solid black line is the preservation rate and grey shaded regions represent the associated uncertainty. Vertical green bars represent the main events discussed in the text: VWAE, Valanginian–Weissert Anoxia Event; OAE‐1a, 1b, 1d, 2, Oceanic Anoxia Event 1a, 1b, 1d, 2; K/Pg, Cretaceous–Palaeogene Extinction Event; PETM, Paleocene–Eocene Thermal Maximum; EECO, Early Eocene Climatic Optimum; MECO, Middle Eocene Climatic Optimum; EOT, Eocene–Oligocene Transition; OMT, Oligocene–Miocene Transition.

**Fig. 2:**
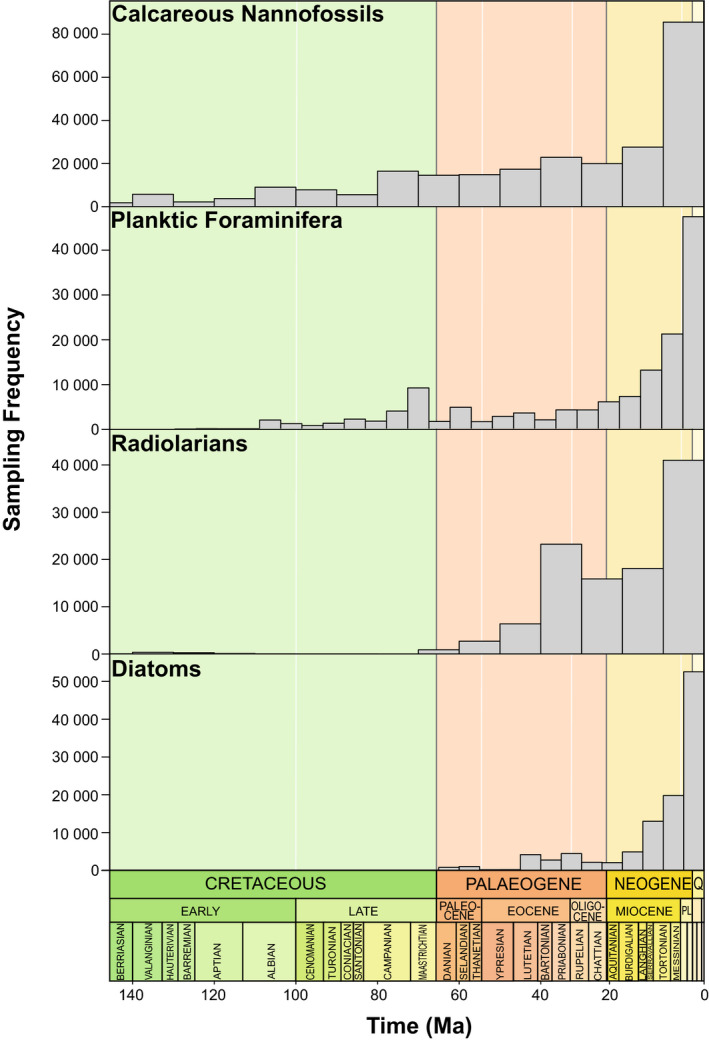
The number of occurrences of each microfossil group from 150 Ma to the Neogene in the NSB database, indicating sampling frequency through time.

Preservation rates tend to increase towards the present day (Fig. 1), however, there are notable deviations in calcareous groups. Foraminifera have relatively high preservation rates consistently throughout the Cretaceous with the lowest preservation rates in the Cenozoic at 60–20 Ma, while nannofossils have intermittent peaks of preservation from 150–60 Ma before steadily increasing towards the Recent. Sampling frequency of all four fossil groups improves towards the present day (Fig. 2). Radiolarians have extremely low sampling rates in the Mesozoic, prior to the occurrence of diatoms in the NSB. Nannofossils are the most sampled group, notably over 80 000 occurrences recorded in the Pliocene and Pleistocene. Nannofossil observations from the Cretaceous to the Recent number 280 934, followed by foraminiferans (157 958), radiolarians (116 527) and diatoms (114 845).

### Model selection

Non‐homogeneous Poisson process (NHPP) preservation models were preferred for all microfossil groups, except diatoms (for which a time‐variable Poisson process (TPP) model was preferred; Table 2). For each microfossil group, the results presented below are from the respective best fitting model (incorporating taxic preservation rate heterogeneity).

**Table 2:** Taxon‐specific selection of the preservation model for each PyRate analysis using maximum likelihood.

PyRate Run	HPP		NHPP		TPP	
	AICc	δAIC	AICc	δAIC	AICc	δAIC
Calcareous microfossil groups	1266659.0	43557.3	**1223101.2** *	–	1235929.0	12827.4
Siliceous microfossil groups	610008.2	18433.2	**591574.9** *	–	594251.4	2676.5
Foraminifera	349132.1	9439.3	**339692.8** *	–	345945.1	6252.3
Nannofossils	908162.4	34773.7	**873388.7** *	–	887865.2	14476.5
Radiolarians	425443.9	17520.6	**407923.3** *	–	410398.7	2475.4
Diatoms	178146.1	11608.4	177212.4	10674.7	**166537.7** *	–

Corrected Akaike Information Criterion (AICc) represents the AIC adjusted for small sample size. δAIC represents the difference in AIC from the statistically best model (highlighted in **bold**). Significance of the best model is indicated.

* Significance at *p* < 0.01.

### The Cretaceous and the K/Pg

#### Calcareous microfossils

**Fig. 3:**
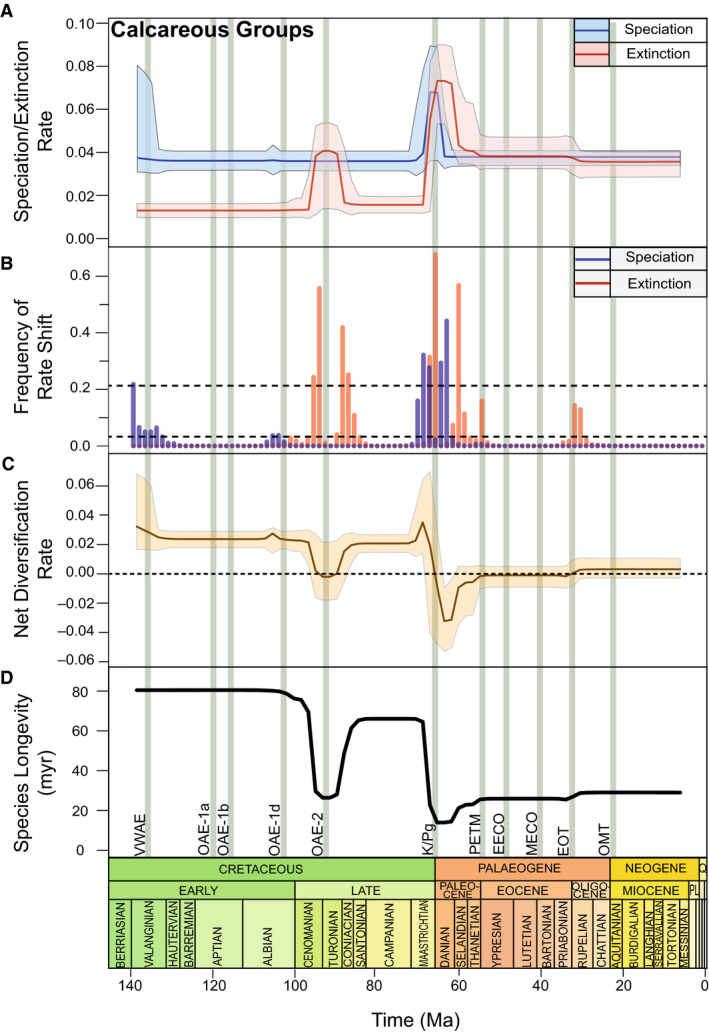
Calcareous microfossil diversification dynamics from 140–5 Ma. Plot showing the combined diversification rates (speciation/extinction events lineage^−1^Ma^−1^) of planktic foraminiferans and calcareous nannofossils. The solid rate lines represent the mean diversification rate, while the shaded area represent the 95% credibility interval (CI). The vertical green bars represent the events discussed in this study from the Cretaceous to the Neogene (see Fig. 1). A, speciation and extinction rates in calcareous microfossils. B, frequency of rate shift plot showing the significance of extinction and speciation rate change; PyRate is designed to generate a frequency of rate shift plot that corresponds to speciation and extinction plots; frequency of rate shifts are calculated using prior probabilities of rate shifts through time to compute posterior sampling frequencies; dashed lines indicate statistical support and are determined using standard log‐Bayes Factor (BF) thresholds; the lower line represents a BF = 2 while the top line represents a BF = 6, suggesting a positive or strong signal respectively (Kass & Raftery 1995). C, net diversification of the two fossil groups from 140–5 Ma; PyRate generates net diversification plots by subtracting extinction rates from speciation rates through time. D, mean species longevity of the two fossil groups.

**Fig. 4:**
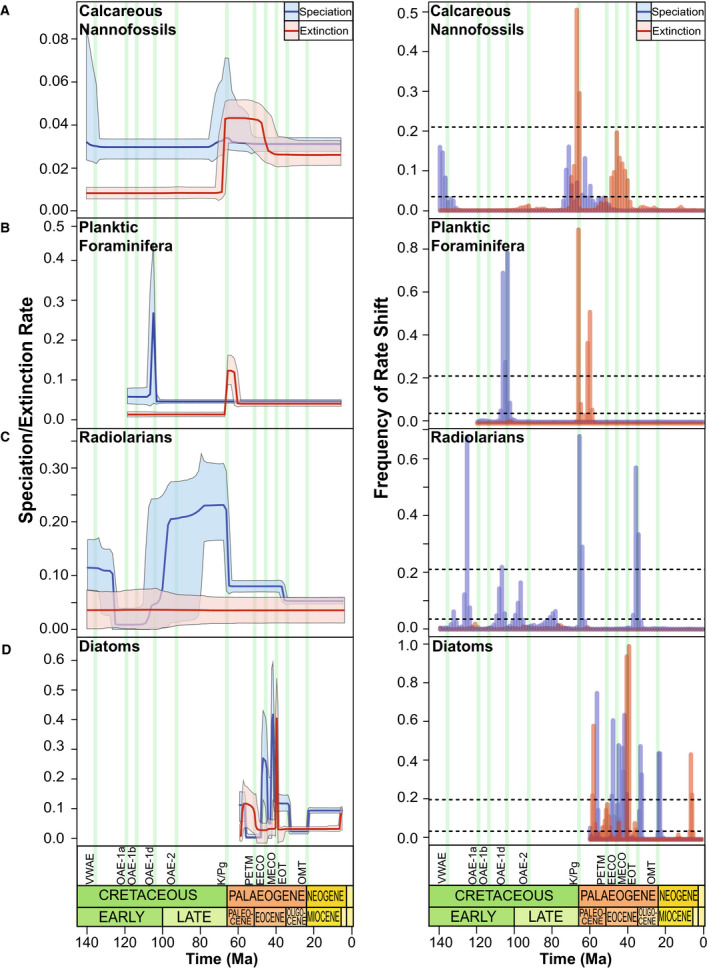
Extinction and speciation rates (left) of the four individual fossil groups with their corresponding frequency of rate shift plot (right) (speciation/extinction events lineage^−1^Ma^−1^). Solid rate lines represent the mean diversification rate; shaded area represents the 95% credibility interval (CI). Vertical green bars represent the events discussed (see Fig. 1). Dashed lines on the frequency of rate shift plots represent log‐Bayes Factor thresholds (see Fig. 3). A, calcareous nannofossil diversification dynamics. B, planktic foraminiferan diversification dynamics. C, radiolarian diversification dynamics. D, diatom diversification dynamics.

Following an initial decline in nannofossils at *c*. 135 Ma, their speciation and extinction rates remain stable through much of the Early Cretaceous (Figs 3A, B, 4A). Foraminifera experience a substantial but abrupt pulse (*c*. 2 myr) of rapid speciation around 105 Ma (Fig. 4B), shortly after the earliest records in this dataset. A more prolonged period of significantly higher rates of extinction (0.043 lineage^−1^Ma^−1^; 95% CI: 0.017 to 0.055 lineage^−1^Ma^−1^) in calcareous microfossils (BF > 6; Fig. 3B) between 95–90 Ma is associated with an overall net negative diversification rate (−0.002 lineage^−1^Ma^−1^; 95% CI: −0.010 to 0.005 lineage^−1^Ma^−1^) and a drastic decrease in species longevity (Fig. 3C, D). While net diversification rates return to prior levels, species longevity is reduced for the remainder of the Late Cretaceous until a brief spike in diversification before the K/Pg (Fig. 3), predominantly in the nannofossils (Fig. 4A).

The largest decline in calcareous diversification in the whole sequence occurs at the K/Pg: extinction rates peak at 66 Ma (0.07 lineage^−1^Ma^−1^; 95% CI: 0.052–0.09 lineage^−1^Ma^−1^; Fig. 3A, B) and mean species longevity declines to its lowest overall level (*c*. 15 myr; Fig. 3D). Both foraminiferans and nannofossils are substantially affected (approximately 14‐fold and 5‐fold increases in mean extinction rates respectively) with very strong support for a change in rates (BF > 6; Figs 3A, B, 4A, B). Speciation rate increases across the boundary, simultaneously with increases in speciation rate within nannofossils (Fig. 4A).

#### Siliceous microfossils

**Fig. 5:**
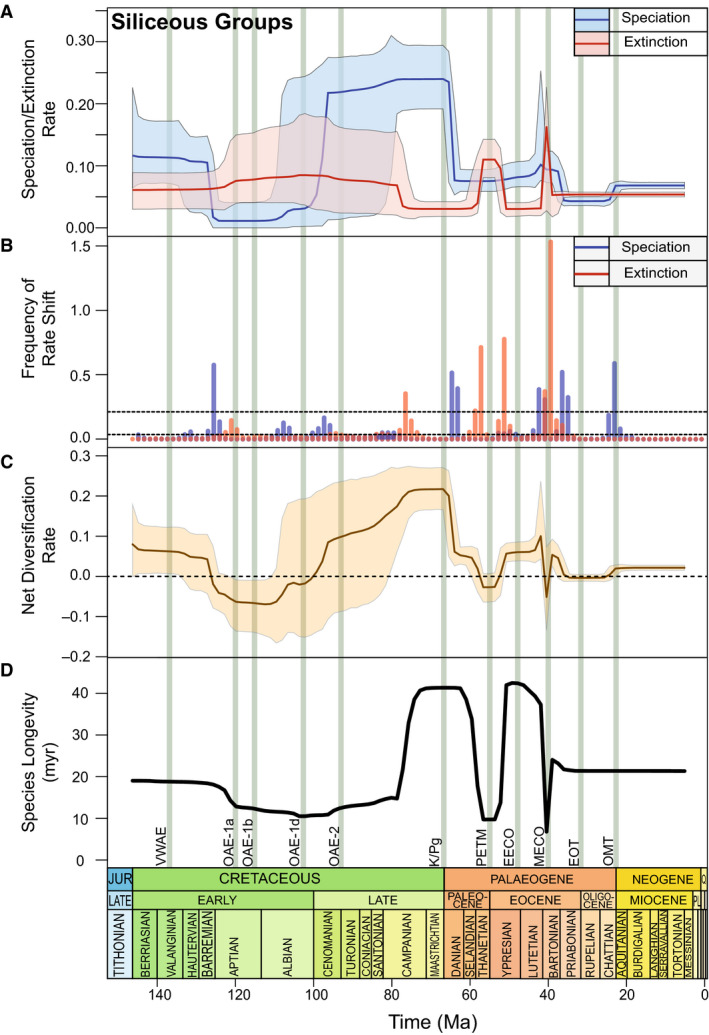
Siliceous microfossils diversification dynamics from 150–5 Ma, showing the combined diversification rates of radiolarians and diatoms (speciation/extinction events lineage^−1^Ma^−1^). Solid rate lines represent the mean diversification rate; shaded area represents the 95% credibility interval (CI). Vertical green bars represent the events discussed (see Fig. 1). A, speciation and extinction rates in siliceous microfossils. B, frequency of rate shift plot showing extinction and speciation rate change; dashed lines represent log‐Bayes Factor (BF) thresholds (see Fig. 3). C, net diversification of siliceous microfossils. D, mean species longevity of siliceous microfossils.

There are no Mesozoic diatom data in NSB and consequently the siliceous groups are represented only by radiolarian data during the Cretaceous and K/Pg. Speciation rate is larger than extinction rate at the beginning of the record (140 Ma) at 0.12 lineage^−1^Ma^−1^ (95% CI: 0.06–0.23 lineage^−1^Ma^−1^; Fig. 5A, B) but gradually declines until a substantial decrease in mean speciation rate (0.10 lineage^−1^Ma^−1^) shifts at *c*. 125 Ma. Net diversification rate falls below 0.0 lineage^−1^Ma^−1^ (95% CI: −0.08 to +0.08 lineage^−1^Ma^−1^; Fig. 5C) and continues to decline, with a corresponding reduction in mean species longevity (*c*. 13 myr; Fig. 5D) by *c*. 120 Ma. Increasing extinction rates further reduce net diversification to −0.08 lineage^−1^Ma^−1^ (95% CI: −0.17 to −0.01 lineage^−1^Ma^−1^) by 115 Ma (Fig. 5A, B). In the early Late Cretaceous, speciation rates increase rapidly while, coupled with a corresponding gradual decrease in extinction rates, net diversification continues to increase throughout the Late Cretaceous to reach a peak at *c*. 76 Ma (Fig. 5A, B). Species longevity increases substantially in the latest Late Cretaceous to 40 Ma (Fig. 5D). Extinction rates remain constant across the K/Pg, however, speciation rates in radiolarians crash to about one‐third of prior levels (0.22 lineage^−1^Ma^−1^ to 0.07 lineage^−1^Ma^−1^; Fig. 4C).

### The Cenozoic

#### Calcareous microfossils

After the K/Pg, net diversification continues to decline until *c*. 63 Ma (Fig. 3C). This is concurrent with the origination of planktic foraminifer *Praemurica inconstans* yet remains negative until the onset of the Eocene. Extinction rates in nannofossils remain elevated for *c*. 16 myr at 0.045 lineage^−1^Ma^−1^ (95% CI: 0.030–0.055 lineage^−1^Ma^−1^) and remain higher than speciation rates until *c*. 40 Ma (Fig. 4A), whereas foraminiferal extinction rates are elevated for only *c*. 6 myr but at *c*. 0.12 lineage^−1^Ma^−1^ (95% CI: 0.09–0.16 lineage^−1^Ma^−1^) following the K/Pg (Fig. 4B). Mean species longevity of calcareous microfossils does not recover to its levels in the Late Cretaceous, increasing by *c*. 12 myr in the Palaeogene (Fig. 3D). Net diversification of calcareous groups is stable until *c*. 33 Ma when it increases above 0 due to a decline in extinction rate (Fig. 3C). Nannofossils and foraminiferans do not display significant extinction rate shifts in the Oligocene and Miocene (BF < 2; Fig. 4A, B).

#### Siliceous microfossils

At *c*. 64 Ma, mean speciation rates drop abruptly (Figs 5A, B) by 0.15 lineage^−1^Ma^−1^ and mean net diversification rate declines by 0.19 lineage^−1^Ma^−1^ (Fig. 5C). Species longevity erratically varies by over 30 myr between the later Paleocene and middle Eocene (Fig. 5D) associated with significant increases in the extinction rate in two pulses in diatoms (*c*. 46 Ma and *c*. 42 Ma, BF > 6; Figs 4D, 5A, B) that causes negative net speciation at 56 Ma (Fig. 5C). Extinction shifts are not acknowledged in the radiolarian record (Fig. 4C); however, a significant extinction rate shift occurs in the diatom record at 38 Ma (Fig. 4D). Simultaneously siliceous group species longevity declines to its lowest value of *c*. 6 myr (Fig. 5D). Prior to the Eocene–Oligocene transition (EOT), net diversification rates stabilize at around 0.0 (95% CI: −0.01 to +0.01 lineage^−1^Ma^−1^; Fig. 5C), which lasts until the Palaeogene–Neogene boundary when an increase in mean speciation rates of diatoms drives an increase in net diversification (0.08 lineage^−1^Ma^−1^ and 0.03 lineage^−1^Ma^−1^ increases in speciation and net diversification respectively; Figs 4D, 5C). An increase in extinction rate coincides with the Miocene–Pliocene transition in the diatom record (Fig. 4D).

## Discussion

### Cretaceous anoxia events

Global perturbations of the carbon cycle extensively affected the world’s oceans and their ecosystems. Diverse biotic and geological data provides evidence that Cretaceous oceans were extremely warm and high, and frequently experienced widespread oceanic anoxia events (OAEs) with important evolutionary impacts (Haq 1973; Leckie 1989; Wilson & Norris 2001; Leckie *et al*. 2002). Periodic and transient horizons of distinct carbon‐rich deposits in Cretaceous sediments are clear indicators of OAEs (Gambacorta *et al*. 2016), which indicate widespread changes in ocean geochemistry (Jenkyns 2010), sea levels, ocean circulation and vertical stratification (Leckie *et al*. 2002). These events caused widespread shifts in microfossil preservation and diversification dynamics (Lowery *et al*. 2020).

#### Valanginian–Weissert Anoxia Event (VWAE)

The calcareous nannofossil speciation decline at *c*. 135 Ma (Figs 3A–B, 4A) coincides with a positive carbon isotope excursion (CIE), detected during the late Valanginian (Weissert 1989; Lini *et al*. 1992; Wortmann & Weissert 2000). Widespread eutrophication (Bottini *et al*. 2018) associated with continental break‐up, basalt flooding and increased crustal production (Soua 2016) resulted in increased transport and nutrification triggering a ‘nannoconid decline’ at the onset of the OAE (Mattioli *et al*. 2014). Large error margins associated with the rate change in nannofossils at 135 Ma (Fig. 4A), may instead represent a false speciation rate shift due to truncated maximum and minimum sample ages in the dataset (Silvestro *et al*. 2014*b*). However, nannofossil preservation reaches its highest value of the Cretaceous during this timeframe (Fig. 1) and therefore probably presents a representative shift in nannofossil diversification. Despite an overall decrease in calcareous diversification rates due to a ‘biocalcification crisis’ (Williams & Bralower 1995; Erba *et al*. 2004), nannofossil genera that indicate ocean fertilization such as *Diazomatolithus* display a marked increase at the VWAE (Barbu & Melinte‐Dobrinescu 2008). This possibly suggests that excess CO_2_ and warm surface waters resulted in higher fertility that promoted selectivity for genera that thrive in eutrophic conditions (Bersezio *et al*. 2002). Planktic Foraminifera are not present in the NSB data until *c*. 120 Ma since the records from the Berriasian to Barremian are scattered stratigraphically and geographically, making interpretations of their evolution difficult (Petrizzo *et al*. 2020). However, Silva & Sliter (1999) and Coccioni *et al*. (2007) noted the appearances of the first planispiral *Globigerinelloides* in the late Valanginian, suggesting that this OAE was not a driving force in foraminiferal evolution. Radiolarians experienced a biotic turnover during this event (Erba *et al*. 2004; Bottini *et al*. 2018), which is not reflected in this study (Fig. 4C), probably due to low preservation rates (Fig. 1) and low sampling frequency (<500 occurrences; Fig. 2) in the middle–late Cretaceous.

#### OAE‐1a

During the early Aptian (*c*. 120 Ma), OAE‐1a, a negative CIE event, caused an interval of organic‐rich black shale deposition in marine sediments from all major ocean basins (Robinson *et al*. 2004; Midtkandal *et al*. 2016; Bauer *et al*. 2019), leading to a decline in siliceous microfossil speciation and diversification rates (Figs 4C, 5A–C). The direct cause of OAE‐1a is still contested, however, increased carbon burial is probably the result of increased volcanism in the south‐west Pacific Ocean and an augmented greenhouse effect (Li *et al*. 2008). Subsequent changes in oceanic circulation patterns (Larson & Erba 1999), rising sea levels (Haq *et al*. 1988; Leckie *et al*. 2002), and enhanced weathering is likely to have caused alterations in biogeochemistry and primary production (Gomes *et al*. 2016). Under these conditions, deeper‐dwelling radiolarians experience species extinctions (Erbacher & Thurow 1997), probably as a result of reduced oxygen saturation or interruptions to vertical ocean stratification creating an unsuitable habitat. It is likely that this led to the last occurrences of deep‐water radiolarian genera *Pantanellium* and *Podobursa* at *c.* 120 Ma (Erbacher & Thurow 1997). Furthermore, a decline in sulphur concentrations and development of ferruginous conditions in the oceans are likely to have promoted anoxia, interrupting planktic micro‐organisms that rely on photosymbiosis (Bauer *et al*. 2019), suggesting that anoxia is significant in controlling siliceous diversity.

Nannofossil diversification does not fluctuate during this event, opposing an expected decline in calcification due to high *p*CO_2_ as seen in modern taxa (Erba & Tremolada 2004). However, it has been demonstrated that OAE‐1a only impacted certain groups (Lowery *et al*. 2020) and coupled with low sampling and preservation during this period (Figs 1, 2), it is probable that calcareous nannofossil decline would not show up in this study. In contrast, other foraminiferan studies demonstrate an increase in extinction rate (Leckie *et al*. 2002), which is not seen here (Fig. 4B). Foraminiferal occurrence data in NSB commences at 120 Ma with exceedingly low preservation rates (Figs 1, 2), hence OAE‐1a is unlikely to be captured in this analysis.

#### OAE‐1b

Characterized by cooling and sea‐level fall, OAE‐1b (late Aptian, *c*. 116 Ma) goes undetected in calcareous microfossils, despite it being recognized as the largest extinction within the Cretaceous for planktic Foraminifera (Fig. 3; Premoli Silva & Sliter 1999; Fraass *et al*. 2015; Lowery *et al*. 2020), and preservation is high during this period (Fig. 1). This may be the result of geographically and stratigraphically limited data during the Albian. Surviving planktic foraminiferans resided in the mixed layer and were typically microperforate species (Petrizzo *et al*. 2020), whereas deeper dwellers were predominantly impacted by reorganization of North Atlantic deep‐water circulation and break down of water column stratification (Huber & Leckie 2011). Consequently, limited Foraminifera sampling of deeper oceanic environments during this period (see Fig. S2) may have led to this extinction event going undetected in this analysis. OAE‐1b presents a dramatic reduction in foraminiferal test size during this time, with average sizes dropping by *c*. 100 µm (Huber & Leckie 2011). Associated with fundamental changes of foraminiferal ultrastructure, large and ornate taxa are shown to disappear in the late Aptian, replaced with simpler and smaller forms (Premoli Silva & Sliter 1999; Leckie *et al*. 2002). Assemblage composition change is associated with prolonged cooling and possible acidification from Kerguelen volcanism (Leckie *et al*. 2002; Petrizzo *et al*. 2020).

OAE‐1b is not reflected by a pronounced change in siliceous diversification since it is unlikely to have recovered from OAE‐1a due to the greenhouse nature of the ocean–climate system (Leckie *et al*. 2002). Net diversification of siliceous microfossils kept stable at *c*. –0.08 lineage^−1^Ma^−1^ (95% CI: −0.15 to −0.01 lineage^−1^Ma^−1^) for the remainder of the Aptian, with elevated extinction rates (Fig. 5A–C) spanning the duration of the mid‐Cretaceous. The poor siliceous preservation rate (Fig. 1) during the Cretaceous may have prevented key biotic events from being detected by PyRate. Additionally, OAE‐1b had a generally regional impact constrained to certain regions such as the North Atlantic and Mediterranean ocean basins, contrasting the global distribution observed at OAE‐1a (Premoli Silva & Sliter 1999).

#### OAE‐1d

The late Albian to early Cenomanian oceanic anoxic event (*c*. 104–99.5 Ma) represents a global interval of black shale deposition, distinguished by a moderate (0.5–2‰) positive carbon isotope (δ^13^C) excursion (Bralower *et al*. 1993; Leckie *et al*. 2002; Watkins *et al*. 2005; Meyers *et al*. 2006; Giorgioni *et al*. 2012). OAE‐1d has been proposed as an interval of global warming, sea‐level rise, and changes in water column stratification due to widespread oxygen deficiency (Watkins *et al*. 2005; Rodríguez‐Cuicas *et al*. 2019; Petrizzo *et al*. 2020), leading to an evolutionary turnover of planktic Foraminifera (Fraass *et al*. 2015). A significant radiation of planktic Foraminifera is observed at *c*. 105 Ma (Fig. 4B), associated with the origination of keeled, deep‐dwelling, and morphologically complex taxa due to increased ecospace (Hart 1990). Extinction associated with a turnover is not reflected in Foraminifera results (Fig. 4B), although occurrences of both *Ticinella* and *Bitticinella*, which developed during OAE‐1b, cease at *c*. 103 Ma (Fraass *et al*. 2015). New mixed layer genera originated, including *Paracostellagerina* and *Planomalina* (Petrizzo *et al*. 2008), suggesting they were speciating to occupy the ecological niche space, vacated by mixed‐layer genera which disappeared during OAE‐1b (Lowery *et al*. 2020). OAE‐1d is enigmatic and intermittent stratification interruptions strongly influenced anoxia in bottom waters, initiating a shift from calcareous to siliceous deposition (Gambacorta *et al*. 2016). Formation of a pycnocline (Tiraboschi *et al*. 2009) would have destabilized calcium carbonate and established an oxygen minimum zone, initiating a loss of deep‐sea habitats (Erbacher & Thurow 1997). Consequently, it is probable that limited occurrence data during this period, especially from pelagic ocean basins, resulted in an extinction event going undetected in Foraminifera.

Eutrophic conditions, rising sea levels, and the collapse of density gradients that developed during OAE‐1d probably led to a turnover in Radiolaria (Wilson & Norris 2001; Leckie *et al*. 2002). Radiolarians are at a net negative diversification rate of –0.02 lineage^−1^Ma^−1^ (95% CI: −0.18 to +0.2 lineage^−1^Ma^−1^) at 105 Ma, before steeply increasing towards the Early–Late Cretaceous boundary (Fig. 4C). Erbacher & Thurow (1997) described the disappearance of 15 radiolarian taxa and origination of 12 new taxa. The abiotic events at the Albian–Cenomanian boundary include an eustatic maximum, which may have allowed radiolarians to diversify precipitously due to a rapid expansion of new niches and increased temperatures (O’Dogherty & Guex 2002; Wang *et al*. 2019). This long‐term sea‐level rise, in addition to oxygen deficiency in bottom waters, is likely to have caused increased marine productivity, upwelling of nutrient‐rich waters, and controlled the shift from calcareous to siliceous deposition, making trends in sea level and eutrophic conditions key factors in radiolarian diversification.

#### OAE‐2

The Cenomanian–Turonian boundary (*c*. 94 Ma) marks one of the most widespread carbon cycle perturbations of the Phanerozoic Eon (Turgeon & Creaser 2008; Clarkson *et al*. 2018). Oceanic anoxia increased globally by at least a factor of three compared to the Recent (Montoya‐Pino *et al*. 2010); this, coupled with high atmospheric CO_2_ and rapid warming due to the eruption of the Caribbean large igneous province (LIP), affected the biota (Leckie *et al*. 2002; Lowery *et al*. 2020). Previous studies have generally shown a stepwise extinction in Radiolaria (Erbacher & Thurow 1997; Lowery *et al*. 2020); our results, however, show speciation rates in siliceous microfossils continuing to increase following OAE‐1d (Fig. 5A, B), and net diversification exceeding 0 lineage^−1^Ma^−1^ by *c*. 95 Ma (Fig. 5C). Warmer temperatures during OAE‐2 triggered rapid pulses of terrigenous nutrients weathered from LIPs (Kuroda *et al*. 2007), which may in turn have promoted an increase in oceanic silica saturation (Jenkyns 2010). During OAE‐2, climate changes were influential on deep circulation dynamics (Gambacorta *et al*. 2016). Warm and saline bottom waters triggering the vertical advection of nutrients and expansion of the oxygen minimum zone are likely to have created favourable conditions for the origination of new radiolarian taxa (Huber *et al*. 2002; Musavu‐Moussavou *et al*. 2007; Wang *et al*. 2019). The vertical distribution and ecological preferences of radiolarians are vitally important to the reported net diversification (Fig. 5C), since deeper dwellers tended to go extinct whereas those in surface waters and shallower depths speciated and increased in diversity (Wang *et al*. 2019). Anoxic bottom waters and warm conditions typically promote high preservation potential in siliceous sediments due to a lack of bioturbation to regenerate dissolved silica back into the water column (e.g. Kidder & Tomescu 2016). However, the number of occurrences (Fig. 2) in NSB within siliceous groups during this period may be responsible for an extinction event going undetected.

Calcareous microfossils depict an opposing trend with a significant increase in extinction rate commencing at *c*. 95 Ma (Fig. 3A–C). Breakdown of vertical density gradients during the later Cenomanian may have contributed to extinctions within deeper‐dwelling Foraminifera, such as *Globigerinelloides bentonensis* (Leckie *et al*. 2002). Evidence of declining calcareous nannofossils diversity across the Cenomanian–Turonian boundary (Lowery *et al*. 2020) also contributed to the decreased calcareous diversification rates. Calcareous species longevity simultaneously plummets by over 40 myr (Fig. 3D), probably influenced by the dominance of short‐lived, smaller, and opportunistic taxa able to tolerate low oxygen conditions (Coccioni & Galeotti 2003). High‐nutrient waters promoted the proliferation of the foraminiferal genus *Schackoina*; it is likely that the elongated and ornamented chambers were important to maximize oxygen and nutrient uptake (Georgescu 2012). At *c*. 90 Ma, diversity and mean species longevity begins to recover but they do not reach levels prior to the elevated extinction. This would suggest that the anoxic event had a long‐lasting impact on calcareous populations, with net diversification in decline for *c*. 5 myr. Gradual yet not complete recovery of foraminiferal assemblages is also noted by Coccioni & Galeotti (2003), resulting from a well‐developed oxygen minimum zone. Despite the anoxia event lasting only *c*. 440 000 years, habitat loss and mass extinction resulted in severe assemblage disruption (Pogge Von Strandmann *et al*. 2013) and deterioration of ecological space (Parente *et al*. 2008).

### Cretaceous–Palaeogene extinction event

The largest and most highly significant extinction rate increase of the calcareous record occurs at *c*. 66 Ma with a precipitous decline in net diversification rate (Fig. 3A–C). The extinction in both terrestrial and marine realms immediately before and across the Cretaceous–Palaeogene boundary appears to have been driven primarily by the Chicxulub impact (Alvarez *et al*. 1980; Schulte *et al*. 2010; Vellekoop *et al*. 2016; Hull *et al*. 2020). An increase in extinction rate is seen to occur in both calcareous nannofossils and planktic foraminiferans (Fig. 4A, B) with the largest impact observed in planktic foraminiferans (*c*. 0.14 lineage^−1^Ma^−1^ increase), as *c*. 95% of taxa within planktic foraminiferans go extinct across the K/Pg caused by incredibly volatile and hostile conditions (Arenillas *et al*. 2016). Aerosols, soot from wildfires, and ejecta sparked a short‐impact winter (Wolbach *et al*. 1990; Tabor *et al*. 2020), reducing climate forcing and ceasing photosynthetic activity (Vellekoop *et al*. 2014). In turn, pelagic stratification broke down causing the mixed layer to plummet from its normal depth of *c*. 100–150 m to >2500 m (Brugger *et al*. 2017). The combined impact of these stressors promoted high extinction and low speciation rates in planktic foraminiferans. Nannofossils display both high extinction and high speciation rates (Fig. 4A), reflected in the calcareous record (Fig. 3A, B). Following the short‐term cooling, aerosols and vaporized carbonates resulted in severe warming (Kring 2007). Enhanced ocean mixing and additional nutrients from settling out ejecta, caused increased nutrient transport from the deep ocean to the surface, and promoted primary production (Brugger *et al*. 2017). Consequently, it is likely that aberrant, opportunistic ‘disaster taxa’ originated in regional blooms shortly after the impact in calcareous groups (Arenillas *et al*. 2018). The pathway the biota took to recover from the mass extinction, and precisely how to define ‘recovery’, is an active subject of current research (e.g. Hull *et al*. 2011; Schueth *et al*. 2015; Lowery *et al*. 2018). Corresponding with species longevity plummeting, these taxa would have been short‐lived, colonizing new morphospace, before diversity began to increase again, resulting in high turnover rates (Lowery & Fraass 2019). Net diversification in calcareous groups takes *c*. 3–4 myr to begin to recover (Fig. 3C). This is concurrent to the re‐establishment of planktic foraminifer photosymbiosis at *c*. 63.5 Ma (Norris 1996; Birch *et al*. 2012). The recorded recovery, however, does not appear to be globally synchronous (Hull & Norris 2011). Danian taxa tended to be unusually small (<150 µm; Gallala *et al*. 2009) since smaller, more angular forms provided a selective advantage for harbouring symbionts in the first photosymbiotic taxa (Birch *et al*. 2012). Deep‐sea transfer of organic matter via the biological pump was severely interrupted (Coxall *et al*. 2006) inhibiting deep‐sea carbonate sedimentation (Peters *et al*. 2013), but evidence of new originations suggests that it was less severely impacted and for less time than previously thought (Birch *et al*. 2012). Extinction remains high in nannofossils well into the Cenozoic, taking 10 myr to reach pre‐extinction diversity (Alvarez *et al*. 2019). This crisis interval is dominated by short‐lived ‘bloom’ taxa such as *Futuyania* and *Prinsius* that thrive in eutrophic conditions. These are replaced by oligotrophic taxa as the climate warms and global thermal stratification increases into the Paleocene (Hilting *et al*. 2008). Shallow, oligotrophic taxa were increasingly uncompetitive and the most successful in the Paleocene, despite experiencing some of the highest extinction rates at the boundary (Jiang *et al*. 2010), perhaps suggesting the simultaneous significant speciation and extinction rates (Fig. 4A). High extinction and speciation rates across the K/Pg in calcareous fossil groups demonstrate that time is required to rebuild ecological niche space to promote novel radiations and increasingly complex morphologies to promote diversification (Lowery & Fraass 2019), though the durations of these radiations are different in this analysis.

Radiolarians, on the other hand, demonstrate continually high speciation and diversification rates throughout the Late Cretaceous and initially across the K/Pg, while extinction rates remain low (Fig. 5A–C) with species longevity reaching its highest values across the boundary (*c*. 42 myr; Fig. 5D). Previously analysed marine sediments suggest moderately rich radiolarian assemblages, with both common and rare species discovered (Jianbing & Aitchison 2002; Hollis & Strong 2003). Data for radiolarians and other siliceous groups appears poorly represented across the K/Pg (Lowery *et al*. 2020) relative to other parts of their record, although preservation increases at *c*. 65 Ma (Fig. 1). At several sites noted by D’Hondt (2005), concentrations of silica increased at the K/Pg and remained high for at least 1–2 myr following the mass extinction, interpreted as an interval of high biosiliceous productivity (Hollis & Strong 2003). Nutrient‐rich waters and cool upwelling zones in the earliest Paleocene are likely to have advanced siliceous plankton speciation (Strong *et al*. 1995). This suggests that SSTs, nutrient input, and ocean circulation patterns are integral in radiations of radiolarians.

### Paleocene and Eocene

Delayed recovery in calcareous taxa following the K/Pg parallels the prolonged extinction in nannofossils, which remain elevated for *c*. 16 myr (Fig. 4A). Net diversification of calcareous taxa gradually increases from *c*. 63 Ma (Fig. 3C) and reaches equilibrium just after the Paleocene–Eocene boundary (56 Ma). The Paleocene–Eocene transition is indicated by an abrupt onset of a negative CIE (Kennett & Stott 1991; Zachos *et al*. 2001*a*) associated with the most significant climatic event of the Cenozoic (Paleocene–Eocene Thermal Maximum (PETM); Zachos *et al*. 2008; Jiang *et al*. 2018) and widely used as an analogue for anthropogenic warming (Hönisch *et al*. 2012; Zeebe & Zachos 2013). During the PETM, calcareous microfossils indicate significant rate changes in extinction and minor rate changes in speciation (Fig. 3B), probably a consequence of cold‐water taxon extinctions and speciation among warm‐dwelling taxa (Mutterlose *et al*. 2007). Nannofossil taxa considered proxies of increased SST (e.g. *Discoaster araneus*, *Rhomboaster cuspis* and *Tribrachiatus bramlettei*) originated between 56 and 55 Ma (Erba & Tremolada 2004; Mutterlose *et al*. 2007). Conversely, low‐latitude taxa were dramatically impacted by the PETM (e.g. losses within *Fasciculithus*). Dissolution linked to calcification depletion from increased CO_2_ and subsequent acidification caused cold water taxa to suffer (Raffi *et al*. 2005). Despite the ecological response at the PETM, Gibbs *et al*. (2006) noted that the rate of turnover was relatively modest and both cool‐water and warm‐water taxa appeared and disappeared making significant extinctions quite rare (Fig. 4A). As a result, the main impact of the PETM was the rapid change in evolutionary turnover as opposed to an individual environmental factor (Gibbs *et al*. 2006), promoting the high latitude extinctions of taxa living at their ecological limits. Planktic foraminiferan diversity increased overall (Fig. 4B; Ezard *et al*. 2011; Fraass *et al*. 2015; Lowery *et al*. 2020). Acidification at the PETM is of the same magnitude as the K/Pg extinction (Penman *et al*. 2014), yet diversification increases overall; indicating that acidification is an unlikely main control on calcareous extinctions (Ridgwell & Schmidt 2010; Hönisch *et al*. 2012), while SST fluctuations thus potentially impact calcareous diversity more. Species longevity decreases across the PETM (Fig. 3D), owing to the origination and subsequent extinctions of short‐lived excursion taxa such as *Morozovella allisonensis* (Kelly *et al*. 1998; Bralower & Self‐Trail 2016).

Following the initial decline after the K/Pg, siliceous extinction rates increase significantly (Fig. 5A–C), and species longevity declines (Fig. 5D) simultaneous to the onset of the PETM. Siliceous diversity throughout the Palaeogene is dynamic, probably resulting from several turnover intervals (Oreshkina 2012) corresponding to significant changes in diatoms (Fig. 4D). Previous studies have noted a turnover in siliceous groups with warm‐water taxa migrating poleward (Liu *et al*. 2011; Oreshkina & Radionova 2014), an extinction of cold‐water taxa, and a rapid proliferation of tropical siliceous taxa across the PETM (Mitlehner 1996). It is likely that these previously seen results are not detected here due to the weakness of the silicate record in this interval, reflected in low preservation and sampling rates (Figs 1, 2). Poor preservation is a known feature of silicate records studies (Oreshkina 2012), and a potential avenue for work moving forward.

After a *c*. 3 myr negative perturbation in siliceous taxa diversification, speciation begins to increase. The rise in biodiversity at *c*. 52–50 Ma has been connected to the Early Eocene Climatic Optimum (EECO; Oreshkina 2012). The EECO has been causally linked to high atmospheric CO_2_ levels thought to correspond to intensified volcanic emissions (Zachos *et al*. 2008), though the extent of these high concentrations is poorly constrained (e.g. Yapp 2004), and the impacts of EECO appear regionally disparate. Extreme changes in regional temperatures and changes in chemical weathering (Zachos *et al*. 2008), are likely to have promoted biosilica depositions in the Arctic (Barron *et al*. 2015). An alternative suggestion is that the Tasmanian Gateway started opening at *c*. 50 Ma, terminating the EECO and leading to an abrupt biosilica deposition (Bijl *et al*. 2013); supported by tectonic studies of seafloor spreading (Livermore *et al*. 2007). Following the EECO, increases in speciation rate occur in two stages in the diatom record with spikes at *c*. 46 Ma and *c*. 42 Ma. These speciation events coincide with the middle Eocene cooling trend and Middle Eocene Climatic Optimum (MECO) respectively (Barron *et al*. 2015). Middle Eocene cooling driven by obliquity cycles initiated extensive diatom deposition in the Atlantic Ocean (Vahlenkamp *et al*. 2018), reflected here (Figs 4D, 5C). Evidence suggests that *Synedropsis*, a genus present at *c*. 47–46 Ma, are sea‐ice dependent and regular melting episodes promoted silica‐rich water, hence increased speciation rates (Stickley & Koc 2009; Stickley *et al*. 2009). Transient warming at *c*. 40 Ma interrupting the long‐term Eocene cooling trend characterizes the MECO (Barron *et al*. 2015), coinciding with a spike in siliceous diversification and diatom speciation rate. Supporting the results of Witkowski *et al*. (2014), high diatom and radiolarian accumulation rates are observed, resulting from increased nutrient supply and subsequent eutrophication from elevated SSTs.

During the Eocene, global carbonate production was in decline, controlled by eccentricity‐scale changes resulting in sudden dissolution episodes (Rivero‐Cuesta *et al*. 2019). However, this was only recorded regionally (Arimoto *et al*. 2020) and consequently not picked up by these analyses. Bleaching was evident in several symbiont‐bearing planktic foraminiferans due to the warming (Edgar *et al*. 2013), yet net diversification rates remain constant within both calcareous fossil groups, despite a fluctuating Eocene climate and highly variable CCD (Fig. 4A, B; Lyle *et al*. 2005). Siliceous diversity peaked during the MECO warming which abruptly ended at *c*. 38–39 Ma as oxygenation recovered and SST decreased (Bohaty *et al*. 2009). Rapid reoxygenation is likely to have initiated a breakdown in water column stratification resulting from decreased fresh‐water input at the cessation of the MECO (Boscolo Galazzo *et al*. 2013), concurrent with a significant change in siliceous diversity. At 38 Ma, siliceous groups experience an abrupt yet short‐lived decline in net diversification (Fig. 5C) and species longevity (Fig. 5D). A decreased sea level and a widespread deep‐sea hiatus occurring at *c*. 38.5 Ma has been put forward as the explanation for a decline in diatom abundance (Wade & Kroon 2002). Discourse as to the trigger of this event is still ongoing. It has been proposed as the outcome of the opening of Drake Passage and Tasman Gateway (Egan *et al*. 2013) or the onset of the Northern Component Water (Borrelli *et al*. 2014), both initiating changes in ocean circulation patterns and influencing the diversity of siliceous taxa. This perturbation is temporally short and siliceous net diversification increases sharply after, paralleled with recorded increases in opal deposition due to increased upwelling (Diekmann *et al*. 2004).

### Eocene–Oligocene Transition

The descent into an icehouse regime continued through the Eocene and a continental ice sheet developed at the Eocene–Oligocene Transition (EOT; Pearson *et al*. 2008). Ice sheet growth was triggered by orbital forcing controlling CO_2_ concentrations (Coxall & Pearson 2007), leading to SSTs dropping by 3–5°C (Galeotti *et al*. 2016). Hypothesized as a turnover or extinction event in all fossil groups, it was expected that EOT diversity would reflect a move to cold‐water taxa dominance while earlier Paleocene–Eocene taxa became extinct. The EOT is typically described as a large extinction in calcareous taxa, due to decreased *p*CO_2_ and subsequent cooling (Wade & Pearson 2008; Lloyd *et al*. 2012*b*; Lowery *et al*. 2020), while siliceous taxa undergo a turnover, with diatoms gaining *c*. 30 new species (Lazarus *et al*. 2014; Barron *et al*. 2015). In contrast to these previous findings, we show a significant shift in extinction rate (Fig. 3B) resulting in a slight increase in diversification in calcareous taxa (Fig. 3C) and a decrease in diversification in siliceous groups (Fig. 5C), though the latter appears to start prior to the EOT. The CCD during the early Oligocene deepened (>1 km) within the tropical Pacific, roughly concurrent with ice sheet growth and sea‐level decline, as deep‐water deposition went from opal‐rich to carbonate‐rich sediments (Coxall *et al*. 2005), thus increasing carbonate preservation potential. These changes, however, are not reflected here.

The Eocene–Oligocene boundary is indicated by the rapid extinction of all remaining species within the Hantkeninidae (Wade & Pearson 2008; Houben *et al*. 2012), with the last occurrence in NSB of *Hantkenina australis* at 34.6 Ma. The Hantkeninidae extinctions were due to changes in marine stratification and pronounced productivity changes (Pearson *et al*. 2008). Despite evidence of small rate shifts within nannofossils (Fig. 4A), these do not exceed the BF thresholds, resulting in calcareous diversification remaining stable from the EOT until the end of the record at 5 Ma. Since radiolarians are well‐preserved and well‐sampled (Figs 1, 2) during the EOT, the decline in siliceous diversification rate may be the result of local radiolarian extinctions with 13 radiolarian taxa disappearing within a 100 000 year period in middle–low latitudes (Kamikuri & Wade 2012). Poor sampling in high latitudes has possibly resulted in speciation going undetected and net diversification subsequently declining.

### Oligocene–Miocene transition

Palaeoclimate records suggest that congruence of eccentricity and obliquity cycles initiated a major transient glaciation at the Oligocene–Miocene Transition (OMT; *c*. 23 Ma; Zachos *et al*. 2001*b*). However, recent research proposes that it may be the result of steadily decreasing CO_2_ levels like the glaciation at the EOT (Greenop *et al*. 2019). Despite its controversial onset, the glaciation during this period is likely to have led to sea‐level decline (Zou *et al*. 2016), and fluctuations of the CCD into the Miocene (Pälike *et al*. 2012). However, these changes do not correlate with increased carbonate species diversity here (Fig. 3C). Increased vertical mixing and intensified ocean circulation led to enhanced latitudinal temperature gradients, promoting an increase in nutrient supply and silicate saturation (Naish *et al*. 2001). Amplified ocean productivity and cool SSTs subsequently drove an increase in diatom species richness (Suto 2006), reflected in Fig. 4D. The important genus *Chaeotoceros* diversifies during this transition, a key development for modern oceans with more than 400 extant species (Suto 2006). Atlantic and Indian Ocean basins in the Miocene have been suggested to have a deep thermocline periodically interrupted by vigorous upwelling, promoting increased productivity at the surface (Smart & Thomas 2006). This upwelling, along with intermittent glacial blanketing of the Antarctic continent, interrupted chemical weathering feedbacks and caused a rise in *p*CO_2_. Warming of up to 2°C is likely to impact foraminiferal assemblages and could have posed a selective advantage for larger taxa. Furthermore, shoaling of the CCD (600 m) at *c*. 18.5 Ma is likely to have caused a ‘carbonate famine’ (Lyle 2003; Pälike *et al*. 2012) and may have promoted a transition to surface‐dwelling, smaller taxa.

From eccentricity triggered Antarctic ice sheet expansion in the middle Miocene (Shevenell *et al*. 2004), to a modest warming interval at the Miocene–Pliocene transition, the Neogene is an important interval in diatom evolution (Lazarus *et al*. 2014). Climate in the Late Miocene and Early Pliocene is likely to have been driven by a combination of *p*CO_2_ fluctuations and tectonically driven partial or complete closure of several oceanic gateways (Lazarus *et al*. 2014), probably leading to an increased extinction rate within diatoms (Fig. 4D). The gradual closure of the Central American gateway at roughly the Miocene–Pliocene transition redirected tropical Atlantic waters northwards, possibly interrupting surface to deep‐sea circulation and diatom productivity (Berger 2007; Butzin *et al*. 2011). Rapid evolutionary turnover in the North Pacific Ocean is also noted due to changes in gyral intensity causing extinction rates to increase to 3 to 4 events per 0.5 myr (Barron & Baldauf 1990; Barron 2003). Following this extinction event, almost 50% of contemporary extant species originated (Lazarus *et al*. 2014), suggesting that diversity–environment relationships during the Miocene and Pliocene were integral to the siliceous diversity of the modern ocean.

### Implications of the microfossil occurrence record

Our results selected the NHPP model for estimating preservation rates in each of the groups analysed (Table 2). This reflects a bell‐shaped distribution of preservation rates through the range of a species, corroborating previous research using a different approach that estimated species’ geographical extent (Liow & Stenseth 2007). While we do not separate geographical effects in our results, it is likely that the drivers of the two models are the same: establishment of a species leads to increased geographical range that supports a higher population. This increased range and population then facilitates preservation and sampling at more sites until species decline reverses this trend (Enquist *et al*. 1995).

The Neptune Database is extensive, comprehensive, and heavily curated, representing one of the highest‐quality palaeontological occurrence datasets. Included fossils are derived from ocean drilling cores, with consistent sampling efforts between samples and groups. The variability in the number of occurrences throughout the interval studied thus represents an accurate picture in fossil preservation bias, both within and between microfossil groups (Figs 1, 2). Despite the extent of this dataset, the results of this study differ from previous studies on the diversity of microfossil groups, missing the fluctuation of nannofossil diversity at OAE‐1a (Erba & Tremolada 2004). The largest change of foraminiferal diversity in the Cretaceous at OAE‐1b (Fraass *et al*. 2015; Lowery *et al*. 2020), and the turnover of siliceous taxa at the PETM (Mitlehner 1996; Liu *et al*. 2011; Oreshkina & Radionova 2014) are three prime examples. The diversification rates for many of the groups reconstructed here are largely static, barring the most major events (Fig. 4). We suggest two reasons behind this inconsistency.

First, preservation biases will change the number of occurrences and the strength of the diversification signal through time, and microfossil preservation will be particularly affected by changes in the marine chemistry (e.g. CCD). While PyRate accounts for variability in preservation (Silvestro *et al*. 2014*a*), periods of reduced occurrences are accompanied by increased uncertainty in the reconstruction of diversification rates (Figs 3, 4, 5). Previous applications of PyRate to the fossil record of vertebrate groups Rhinoceratoidea and marine mammals show increased uncertainty in their reconstruction of diversification rate where occurrences are less frequent (Silvestro *et al*. 2014*a*, 2019). We argue that results produced by PyRate, and its underlying Bayesian methodology, benefit greatly from incorporating and presenting this uncertainty. The Neptune Database has highly precisely dated occurrence data (±0.25 myr, as applied in this study), yet the potential smearing effects described above apparently reduced the otherwise lively diversification record of the microfossils included (particularly calcareous forms; Fig. 4). It is expected that similar effects will apply to other groups, and so these effects should be considered when interpreting the results.

Second, many of the previously identified changes that are not picked up in our analyses are regionally limited or temporally sweep through different areas, for example OAE‐1b and the EECO (Premoli Silva & Sliter 1999; Zachos *et al*. 2008). As we use a global microfossil record, our analyses may thus smooth over all but the most abrupt or widespread events. This highlights the importance of considering the spatiotemporal resolution of occurrence data in analyses (Close *et al*. 2020). We recommend that future research should consider regional variation in diversity signal as well as global trends. In the study of groups that have a relatively poorer fossil record, less frequent sampling, and/or occurrences, applying the relevant resolution is also important.

## Conclusion and future study

Diversity within all microfossil groups fluctuated through time. PyRate tests existing hypotheses associated with major transition periods by ascertaining speciation and extinction rate variations through time. All microfossil groups were uniquely impacted by the different environmental perturbations, but their responses have not been consistent from one extinction to the next. Climatic and tectonic influences appear to be the primary drivers of changes in speciation and extinction (although diversity dependence (Ezard *et al*. 2011) was not considered); temperature, silicate weathering and nutrient input, ocean circulation patterns altered by oceanic gateways, and upwelling zones strongly influence diversification within both calcareous and siliceous groups. Perturbations associated with warm waters, with a coincident deepening of the CCD, often promoted diversification in planktic foraminiferans and calcareous nannofossils, while cooler SSTs, high latitudes and elevated nutrient inputs are associated with radiolarian and diatom diversification. However, opposing behaviours in calcareous and siliceous taxa were occasionally inconsistent; for example, during Cretaceous anoxia events. The EOT results contrast against previous studies of the glaciation event, with little evidence of a biotic turnover; instead net diversification increased unexpectedly in calcareous forms.

PyRate provides a leap forward in quantitative testing of diversification rate changes throughout geological time. In combination with raw fossil data, phylogenetics and palaeoclimate data, PyRate could provide an increasingly vigorous method to quantitatively determine diversification dynamics. However, it is necessary to acknowledge that PyRate does improve its reliability when using a robust dataset. This study therefore highlights the need for higher resolution records of siliceous biota since they are underrepresented in microfossil databases, particularly at high latitudes, whenever possible, as they are integral to analysing glacial intervals. Additionally, extending the record further back in time could resolve discrepancies associated with OAE events. Nonetheless, PyRate provides a novel method within microfossil diversity to statistically tease out speciation and extinction rates, with the potential for further exciting developments. Since major environmental issues affecting the ocean today include rapid onset warming and precipitous increases in carbon dioxide and the subsequent acidification, it is fundamental to study events such as OAEs and the PETM on both regional and global scales. Investigating mass extinctions such as the K/Pg and major transitions in climate such as the EOT provide insights into ecosystem recovery and evolutionary response to major global change. Determining what drives these changes in diversity, assemblage composition, productivity, and selectivity across events, is imperative to improve our understanding of ancient oceans and how our ocean ecosystems might respond today.

## Author contributions

BCM and AJF conceptualized and supervized the project; KMJ carried out the formal analyses, investigation, visualization, and wrote the first draft; all authors contributed to the data curation and methodology. All authors validated and agreed on the final version of the manuscript.

## Supporting information


**Fig. S1**. Sensitivity of PyRate analyses to occurrence age.

**Fig. S2**. Number of species occurrences at different sampling depths.

**Fig. S3**. Latitudinal differences in sampling frequency of microfossil occurrences.

**Data S1**. Example data extraction from Neptune Sandbox Berlin.

**Data S2**. Example PyRate Script used for diversification analysis.

## Data Availability

Data for this study are available on the Neptune Sandbox (https://nsb.mfn‐berlin.de/search/). Code for the analyses and data for this study are available in the Dryad Digital Repository: https://doi.org/10.5061/dryad.2fqz612pk.

## References

[pala12615-bib-0001] Alroy, J. 2010. The shifting balance of diversity among major marine animal groups. Science, 329, 1191–1194. 20813951 10.1126/science.1189910

[pala12615-bib-0002] Alvarez, L. W. , Alvarez, W. , Asaro, F. and Michel, H. V. 1980. Extraterrestrial cause for the Cretaceous‐Tertiary extinction. Science, 208, 1095–1108. 17783054 10.1126/science.208.4448.1095

[pala12615-bib-0003] Alvarez, S. A. , Gibbs, S. J. , Bown, P. R. , Kim, H. , Sheward, R. M. and Ridgwell, A. 2019. Diversity decoupled from ecosystem function and resilience during mass extinction recovery. Nature, 574, 242–245. 31554971 10.1038/s41586-019-1590-8

[pala12615-bib-0004] Andrade, M. , Font, E. , Adatte, T. , Khozyem, H. and Abrajevitch, A. 2018. Magnetic and mercury anomalies at the Paleocene‐Eocene Thermal Maximum (PETM): environmental acidification and the role of volcanism. 20th EGU General Assembly, EGU2018, Proceedings from the conference held 4–13 April, 2018 in Vienna, Austria, p. 1430.

[pala12615-bib-0005] Arenillas, I. , Arz, J. A. , Grajales‐Nishimura, J. M. , Meléndez, A. and Rojas‐Consuegra, R. 2016. The Chicxulub impact is synchronous with the planktonic Foraminifera mass extinction at the Cretaceous/Paleogene boundary: new evidence from the Moncada section, Cuba. Geologica Acta, 14, 35–51.

[pala12615-bib-0006] Arenillas, I. , Arz, J. A. and Gilabert, V. 2018. Blooms of aberrant planktic Foraminifera across the K/Pg boundary in the Western Tethys: causes and evolutionary implications. Paleobiology, 44, 460–489.

[pala12615-bib-0007] Arimoto, J. , Nishi, H. , Kuroyanagi, A. , Takashima, R. , Matsui, H. and Ikehara, M. 2020. Changes in upper ocean hydrography and productivity across the Middle Eocene Climatic Optimum: local insights and global implications from the Northwest Atlantic. Global & Planetary Change, 193, 103258.

[pala12615-bib-0008] Aze, T. , Ezard, T. H. G. , Purvis, A. , Coxall, H. K. , Stewart, D. R. M. , Wade, B. S. and Pearson, P. N. 2011. A phylogeny of Cenozoic macroperforate planktonic Foraminifera from fossil data. Biological Reviews, 86, 900–927. 21492379 10.1111/j.1469-185X.2011.00178.x

[pala12615-bib-0009] Barbu, V. and Melinte‐Dobrinescu, M. C. 2008. Latest Jurassic to earliest Cretaceous paleoenvironmental changes in the Southern Carpathians, Romania: regional record of the late Valanginian nutrification event. Cretaceous Research, 29, 790–802.

[pala12615-bib-0010] Barron, J. A. 2003. Planktonic marine diatom record of the past 18 m.y.: appearances and extinctions in the Pacific and Southern oceans. Diatom Research, 18, 203–224.

[pala12615-bib-0011] Barron, J. A. and Baldauf, J. G. 1990. Development of biosiliceous sedimentation in the North Pacific during the Miocene and early Pliocene. 43–63. *In* Tsuchi, R. (ed.) Pacific Neogene events: Their timing, nature and interrelationship. University of Tokyo Press.

[pala12615-bib-0012] Barron, J. A. , Stickley, C. E. and Bukry, D. 2015. Paleoceanographic, and paleoclimatic constraints on the global Eocene diatom and silicoflagellate record. Palaeogeography, Palaeoclimatology, Palaeoecology, 422, 85–100.

[pala12615-bib-0013] Bauer, K. , Bottini, C. , Katsev, S. , Jellinek, M. , Francois, R. , Erba, E. and Crowe, S. 2019. Ferruginous oceans during OAE1a and the collapse of the seawater sulphate reservoir. Version 1. Earth ArXiv. 10.31223/osf.io/n2b8e

[pala12615-bib-0014] Bé, A. W. H. and Hutson, W. H. 1977. Ecology of planktonic Foraminifera and biogeographic patterns of life and fossil assemblages in the Indian Ocean. Micropaleontology, 369–414.

[pala12615-bib-0015] Berger, W. H. 2007. Cenozoic cooling, Antarctic nutrient pump, and the evolution of whales. Deep Sea Research Part II: Topical Studies in Oceanography, 54, 2399–2421.

[pala12615-bib-0016] Bersezio, R. , Erba, E. , Gorza, M. and Riva, A. 2002. Berriasian–Aptian black shales of the Maiolica formation (Lombardian Basin, Southern Alps, Northern Italy): local to global events. Palaeogeography, Palaeoclimatology, Palaeoecology, 180, 253–275.

[pala12615-bib-0017] Bijl, P. K. , Bendle, J. A. P. , Bohaty, S. M. , Pross, J. , Schouten, S. , Tauxe, L. , Stickley, C. E. , McKay, R. M. , Röhl, U. , Olney, M. , Sluijs, A. , Escutia, C. and Brinkhuis, H. 2013. Eocene cooling linked to early flow across the Tasmanian Gateway. Proceedings of the National Academy of Sciences, 110, 9645–9650. 10.1073/pnas.1220872110 PMC3683727 23720311

[pala12615-bib-0018] Birch, H. S. , Coxall, H. K. and Pearson, P. N. 2012. Evolutionary ecology of Early Paleocene planktonic Foraminifera: size, depth habitat and symbiosis. Paleobiology, 38, 374–390.

[pala12615-bib-0019] Bohaty, S. M. , Zachos, J. C. , Florindo, F. and Delaney, M. L. 2009. Coupled greenhouse warming and deep‐sea acidification in the middle Eocene. Paleoceanography, 24, PA2207.

[pala12615-bib-0020] Borrelli, C. , Cramer, B. S. and Katz, M. E. 2014. Bipolar Atlantic deepwater circulation in the middle‐late Eocene: effects of Southern Ocean gateway openings. Paleoceanography, 29, 308–327.

[pala12615-bib-0021] Boscolo Galazzo, F. , Giusberti, L. , Luciani, V. and Thomas, E. 2013. Paleoenvironmental changes during the Middle Eocene Climatic Optimum (MECO) and its aftermath: the benthic foraminiferal record from the Alano section (NE Italy). Palaeogeography, Palaeoclimatology, Palaeoecology, 378, 22–35.

[pala12615-bib-0022] Bottini, C. , Dieni, I. , Erba, E. , Massari, F. and Weissert, H. 2018. The Valanginian Weissert oceanic anoxic event recorded in central‐eastern Sardinia (Italy). Rivista Italiana di Paleontologia e Stratigrafia, 124, 617–637.

[pala12615-bib-0023] Bown, P. R. , Lees, J. A. and Young, J. R. 2004. Calcareous nannoplankton evolution and diversity through time. 481–508. *In* Thierstein, H. R. and Young, J. R. (eds). Coccolithophores: From molecular processes to global impact. Springer.

[pala12615-bib-0024] Bralower, T. J. and Self‐Trail, J. M. 2016. Nannoplankton malformation during the Paleocene‐Eocene Thermal Maximum and its paleoecological and paleoceanographic significance. Paleoceanography, 31, 1423–1439.

[pala12615-bib-0025] Bralower, T. , Sliter, W. , Arthur, M. , Leckie, M. , Allard, D. and Schlanger, S. 1993. Dysoxic/anoxic episodes in the Aptian‐Albian (Early Cretaceous). 5–37. *In* Pringle , M. S. , Sager , W. W. , Sliter , W. V. and Stein , S. (eds). The Mesozoic Pacific: Geology, tectonics, and volcanism. American Geophysical Union, Geophysical Monograph Series, 77.

[pala12615-bib-0026] Brugger, J. , Feulner, G. and Petri, S. 2017. Baby, it’s cold outside: climate model simulations of the effects of the asteroid impact at the end of the Cretaceous. Geophysical Research Letters, 44, 419–427.

[pala12615-bib-0027] Butzin, M. , Lohmann, G. and Bickert, T. 2011. Miocene ocean circulation inferred from marine carbon cycle modeling combined with benthic isotope records. Paleoceanography, 26, PA1203.

[pala12615-bib-0028] Campbell, S. M. , Moucha, R. , Derry, L. A. and Raymo, M. E. 2018. Effects of dynamic topography on the Cenozoic carbonate compensation depth. Geochemistry, Geophysics, Geosystems, 19, 1025–1034.

[pala12615-bib-0029] de Cárcer, D. A. , Denman, S. E. , McSweeney, C. and Morrison, M. 2011. Evaluation of subsampling‐based normalization strategies for tagged high‐throughput sequencing data sets from gut microbiomes. Applied & Environmental Microbiology, 77, 8795–8798. 21984239 10.1128/AEM.05491-11 PMC3233110

[pala12615-bib-0030] Ceballos, G. , Ehrlich, P. R. , Barnosky, A. D. , García, A. , Pringle, R. M. and Palmer, T. M. 2015. Accelerated modern human–induced species losses: entering the sixth mass extinction. Science Advances, 1, e1400253. 26601195 10.1126/sciadv.1400253 PMC4640606

[pala12615-bib-0031] Chao, A. and Jost, L. 2012. Coverage‐based rarefaction and extrapolation: standardizing samples by completeness rather than size. Ecology, 93, 2533–2547. 23431585 10.1890/11-1952.1

[pala12615-bib-0032] Clarkson, M. O. , Stirling, C. H. , Jenkyns, H. C. , Dickson, A. J. , Porcelli, D. , Moy, C. M. , Pogge von Strandmann, P. A. E. , Cooke, I. R. and Lenton, T. M. 2018. Uranium isotope evidence for two episodes of deoxygenation during Oceanic Anoxic Event 2. Proceedings of the National Academy of Sciences, 115, 2918–2923. 10.1073/pnas.1715278115 PMC5866551 29507196

[pala12615-bib-0033] Close, R. A. , Evers, S. W. , Alroy, J. and Butler, R. J. 2018. How should we estimate diversity in the fossil record? Testing richness estimators using sampling‐standardised discovery curves. Methods in Ecology & Evolution, 9, 1386–1400.

[pala12615-bib-0034] Close, R. A. , Benson, R. B. J. , Saupe, E. E. , Clapham, M. E. and Butler, R. J. 2020. The spatial structure of Phanerozoic marine animal diversity. Science, 368, 420–424. 32327597 10.1126/science.aay8309

[pala12615-bib-0035] Coccioni, R. and Galeotti, S. 2003. The mid‐Cenomanian Event: prelude to OAE 2. Palaeogeography, Palaeoclimatology, Palaeoecology, 190, 427–440.

[pala12615-bib-0036] Coccioni, R. , Silva, I. P. , Marsili, A. and Verga, D. 2007. First radiation of Cretaceous planktonic Foraminifera with radially elongate chambers at Angles (Southeastern France) and biostratigraphic implications. Revue de Micropaléontologie, 50, 215–224.

[pala12615-bib-0037] Coxall, H. K. and Pearson, P. N. 2007. The Eocene–Oligocene transition. 351–387. *In* Williams, M. , Haywood, A. M. , Gregory, F. J. and Schmidt, D. N. (eds). Deep time perspectives on climate change: Marrying the signal from computer models and biological proxies. Geological Society of London.

[pala12615-bib-0038] Coxall, H. K. , Wilson, P. A. , Pälike, H. , Lear, C. H. and Backman, J. 2005. Rapid stepwise onset of Antarctic glaciation and deeper calcite compensation in the Pacific Ocean. Nature, 433, 53–57. 15635407 10.1038/nature03135

[pala12615-bib-0039] Coxall, H. K. , D’Hondt, S. and Zachos, J. C. 2006. Pelagic evolution and environmental recovery after the Cretaceous‐Paleogene mass extinction. Geology, 34, 297–300.

[pala12615-bib-0040] D’Hondt, S. 2005. Consequences of the Cretaceous/Paleogene mass extinction for marine ecosystems. Annual Review of Ecology Evolution & Systematics, 36, 295–317.

[pala12615-bib-0041] Dickens, G. R. , O’Neil, J. R. , Rea, D. K. and Owen, R. M. 1995. Dissociation of oceanic methane hydrate as a cause of the carbon isotope excursion at the end of the Paleocene. Paleoceanography, 10, 965–971.

[pala12615-bib-0042] Diekmann, B. , Kuhn, G. , Gersonde, R. and Mackensen, A. 2004. Middle Eocene to early Miocene environmental changes in the sub‐Antarctic Southern Ocean: evidence from biogenic and terrigenous depositional patterns at ODP Site 1090. Global & Planetary Change, 40, 295–313.

[pala12615-bib-0043] Dinarès‐Turell, J. , Westerhold, T. , Pujalte, V. , Röhl, U. and Kroon, D. 2014. Astronomical calibration of the Danian stage (Early Paleocene) revisited: settling chronologies of sedimentary records across the Atlantic and Pacific Oceans. Earth & Planetary Science Letters, 405, 119–131.

[pala12615-bib-0044] Dutkiewicz, A. , O’Callaghan, S. and Müller, R. D. 2016. Controls on the distribution of deep‐sea sediments. Geochemistry, Geophysics, Geosystems, 17, 3075–3098.

[pala12615-bib-0045] Edgar, K. M. , Bohaty, S. M. , Gibbs, S. J. , Sexton, P. F. , Norris, R. D. and Wilson, P. A. 2013. Symbiont ‘bleaching’ in planktic Foraminifera during the Middle Eocene Climatic Optimum. Geology, 41, 15–18.

[pala12615-bib-0046] Egan, K. E. , Rickaby, R. E. M. , Hendry, K. R. and Halliday, A. N. 2013. Opening the gateways for diatoms primes Earth for Antarctic glaciation. Earth & Planetary Science Letters, 375, 34–43.

[pala12615-bib-0047] Egge, J. K. and Aksnes, D. L. 1992. Silicate as regulating nutrient in phytoplankton competition. Marine Ecology Progress Series, 83, 281–289.

[pala12615-bib-0048] Enquist, B. J. , Jordan, M. A. and Brown, J. H. 1995. Connections between ecology, biogeography, and paleobiology: relationship between local abundance and geographic distribution in fossil and recent molluscs. Evolutionary Ecology, 9, 586–604.

[pala12615-bib-0049] Erba, E. 2004. Calcareous nannofossils and Mesozoic oceanic anoxic events. Marine Micropaleontology, 52, 85–106.

[pala12615-bib-0050] Erba, E. and Tremolada, F. 2004. Nannofossil carbonate fluxes during the Early Cretaceous: phytoplankton response to nutrification episodes, atmospheric CO_2_, and anoxia. Paleoceanography, 19, PA1008.

[pala12615-bib-0051] Erba, E. , Bartolini, A. and Larson, R. L. 2004. Valanginian Weissert oceanic anoxic event. Geology, 32, 149–152.

[pala12615-bib-0052] Erbacher, J. and Thurow, J. 1997. Influence of oceanic anoxic events on the evolution of mid‐Cretaceous radiolaria in the North Atlantic and western Tethys. Marine Micropaleontology, 30, 139–158.

[pala12615-bib-0053] Erwin, D. H. 2001. Lessons from the past: biotic recoveries from mass extinctions. Proceedings of the National Academy of Sciences, 98, 5399–5403. 10.1073/pnas.091092698 PMC33225 11344285

[pala12615-bib-0054] Etienne, R. S. and Apol, M. E. F. 2009. Estimating speciation and extinction rates from diversity data and the fossil record. Evolution, 63, 244–255. 19055680 10.1111/j.1558-5646.2008.00537.x

[pala12615-bib-0055] Ezard, T. H. G. , Aze, T. , Pearson, P. N. and Purvis, A. 2011. Interplay between changing climate and species’ ecology drives macroevolutionary dynamics. Science, 332, 349–351. 21493859 10.1126/science.1203060

[pala12615-bib-0056] Foote, M. 2010. The geological history of biodiversity. 479–510. *In* Bell, M. A. , Futuyma, D. J. , Eanes, W. F. and Levinton, J. S. (eds). Evolution since Darwin: The first 150 years. Sinauer Associates.

[pala12615-bib-0057] Foote, M. , Crampton, J. S. , Beu, A. G. and Cooper, R. A. 2008. On the bidirectional relationship between geographic range and taxonomic duration. Paleobiology, 34, 421–433.

[pala12615-bib-0058] Fordham, B. G. , Aze, T. , Haller, C. , Zehady, A. K. , Pearson, P. N. , Ogg, J. G. and Wade, B. S. 2018. Future‐proofing the Cenozoic macroperforate planktonic Foraminifera phylogeny of Aze & others (2011). PLoS One, 13, e0204625. 30379910 10.1371/journal.pone.0204625 PMC6209145

[pala12615-bib-0059] Fraass, A. J. , Kelly, D. C. and Peters, S. E. 2015. Macroevolutionary history of the planktic Foraminifera. Annual Review of Earth & Planetary Sciences, 43, 139–166.

[pala12615-bib-0060] Galeotti, S. , Deconto, R. , Naish, T. , Stocchi, P. , Florindo, F. , Pagani, M. , Barrett, P. , Bohaty, S. M. , Lanci, L. and Pollard, D. 2016. Antarctic Ice Sheet variability across the Eocene‐Oligocene boundary climate transition. Science, 352, 76–80. 27034370 10.1126/science.aab0669

[pala12615-bib-0061] Gallala, N. , Zaghbib‐Turki, D. , Arenillas, I. , Arz, J. A. and Molina, E. 2009. Catastrophic mass extinction and assemblage evolution in planktic Foraminifera across the Cretaceous/Paleogene (K/Pg) boundary at Bidart (SW France). Marine Micropaleontology, 72, 196–209.

[pala12615-bib-0062] Gambacorta, G. , Bersezio, R. , Weissert, H. and Erba, E. 2016. Onset and demise of Cretaceous oceanic anoxic events: the coupling of surface and bottom oceanic processes in two pelagic basins of the western Tethys. Paleoceanography, 31, 732–757.

[pala12615-bib-0063] Georgescu, M. 2012. Morphology, taxonomy, stratigraphical distribution and evolutionary classification of the schackoinid planktic Foraminifera (late Albian‐Maastrichtian, cretaceous). 1–62. *In* Bailey, D. R. and Howard, S. E. (eds). Deep‐sea: Marine biology, geology and human impact. Nova Publishers.

[pala12615-bib-0064] Gibbs, S. , Bown, P. , Sessa, J. , Bralower, T. and Wilson, P. 2006. Nannoplankton extinction and origination across the Paleocene‐Eocene thermal maximum. Science, 314, 1770–1773. 17170303 10.1126/science.1133902

[pala12615-bib-0065] Giorgioni, M. , Weissert, H. , Bernasconi, S. M. , Hochuli, P. A. , Coccioni, R. and Keller, C. E. 2012. Orbital control on carbon cycle and oceanography in the mid‐Cretaceous greenhouse. Paleoceanography, 27, PA1204.

[pala12615-bib-0066] Gomes, M. L. , Hurtgen, M. T. and Sageman, B. B. 2016. Biogeochemical sulfur cycling during Cretaceous oceanic anoxic events: a comparison of OAE1a and OAE2. Paleoceanography, 31, 233–251.

[pala12615-bib-0067] Greene, S. E. , Ridgwell, A. , Kirtland Turner, S. , Schmidt, D. N. , Pälike, H. , Thomas, E. , Greene, L. K. and Hoogakker, B. A. A. 2019. Early Cenozoic decoupling of climate and carbonate compensation depth trends. Paleoceanography & Paleoclimatology, 34, 930–945. 31598585 10.1029/2019PA003601 PMC6774345

[pala12615-bib-0068] Greenop, R. , Sosdian, S. M. , Henehan, M. J. , Wilson, P. A. , Lear, C. H. and Foster, G. L. 2019. Orbital forcing, ice volume, and CO_2_ across the Oligocene‐Miocene transition. Paleoceanography & Paleoclimatology, 34, 316–328.

[pala12615-bib-0069] Haq, B. U. 1973. Transgressions, climatic change and the diversity of calcareous nannoplankton. Marine Geology, 15, M25–M30.

[pala12615-bib-0070] Haq, B. U. , Hardenbol, J. and Vail, P. R. 1988. Mesozoic and Cenozoic chronostratigraphy and cycles of sea‐level change. 71–108. *In* Wilgus , C. K. , Hastings , B. S. , Posamentier , H. , Van Wagoner , J. , Ross , C. A. and Kendall, C. G. st. C. (eds). Sea‐level changes: An integrated approach. SEPM Special Publication, 42.

[pala12615-bib-1001] Hart, M. B. 1990. Major evolutionary radiations of the planktonic Foraminiferida. 59–72. *In* Taylor, P. D. and Larwood, G. P. (eds). Major evolutionary radiations. Systematics Association Special Volume, **42**. Clarendon Press.

[pala12615-bib-0071] Hilting, A. K. , Kump, L. R. and Bralower, T. J. 2008. Variations in the oceanic vertical carbon isotope gradient and their implications for the Paleocene‐Eocene biological pump. Paleoceanography, 23, PA3222.

[pala12615-bib-0073] Hollis, C. J. and Strong, C. P. 2003. Biostratigraphic review of the Cretaceous/Tertiary boundary transition, mid‐Waipara river section, North Canterbury, New Zealand. New Zealand Journal of Geology & Geophysics, 46, 243–253.

[pala12615-bib-0074] Hönisch, B. , Ridgwell, A. , Schmidt, D. N. , Thomas, E. , Gibbs, S. J. , Sluijs, A. , Zeebe, R. , Kump, L. , Martindale, R. C. and Greene, S. E. 2012. The geological record of ocean acidification. Science, 335, 1058–1063. 22383840 10.1126/science.1208277

[pala12615-bib-0075] Houben, A. J. P. , van Mourik, C. A. , Montanari, A. , Coccioni, R. and Brinkhuis, H. 2012. The Eocene–Oligocene transition: changes in sea level, temperature or both? Palaeogeography, Palaeoclimatology, Palaeoecology, 335–336, 75–83.

[pala12615-bib-0076] Huber, B. T. and Leckie, R. M. 2011. Planktic foraminiferal species turnover across deep‐sea Aptian/Albian boundary sections. Journal of Foraminiferal Research, 41, 53–95.

[pala12615-bib-0077] Huber, B. T. , Norris, R. D. and MacLeod, K. G. 2002. Deep‐sea paleotemperature record of extreme warmth during the Cretaceous. Geology, 30, 123–126.

[pala12615-bib-0078] Hull, P. M. and Norris, R. D. 2011. Diverse patterns of ocean export productivity change across the Cretaceous‐Paleogene boundary: new insights from biogenic barium. Paleoceanography, 26, PA3205.

[pala12615-bib-0079] Hull, P. M. , Norris, R. D. , Bralower, T. J. and Schueth, J. D. 2011. A role for chance in marine recovery from the end‐Cretaceous extinction. Nature Geoscience, 4, 856–860.

[pala12615-bib-0080] Hull, P. M. , Bornemann, A. , Penman, D. E. , Henehan, M. J. , Norris, R. D. , Wilson, P. A. , Blum, P. , Alegret, L. , Batenburg, S. J. and Bown, P. R. 2020. On impact and volcanism across the Cretaceous‐Paleogene boundary. Science, 367, 266–272. 31949074 10.1126/science.aay5055

[pala12615-bib-0081] IPCC In Press. Climate Change 2021: The physical science basis. Contribution of Working Group I to the Sixth Assessment Report of the Intergovernmental Panel on Climate Change. Masson‐Delmotte, V. , Zhai, P. , Pirani, A. , Connors, S. L. , Péan, C. , Berger, S. , Caud, N. , Chen, Y. , Goldfarb, L. , Gomis, M. I. , Huang, M. , Leitzell, K. , Lonnoy, E. , Matthews, J. B. R. , Maycock, T. K. , Waterfield, T. , Yelekçi, O. , Yu, R. and Zhou, B. (eds). Cambridge University Press.

[pala12615-bib-0082] Jenkyns, H. C. 2010. Geochemistry of oceanic anoxic events. Geochemistry, Geophysics, Geosystems, 11, Q03004.

[pala12615-bib-0084] Jianbing, L. and Aitchison, J. C. 2002. Upper Paleocene radiolarians from the Yamdrok mélange, south Xizang (Tibet), China. Micropaleontology, 48, 145–154.

[pala12615-bib-0085] Jiang, S. , Bralower, T. J. , Patzkowsky, M. E. , Kump, L. R. and Schueth, J. D. 2010. Geographic controls on nannoplankton extinction across the Cretaceous/Palaeogene boundary. Nature Geoscience, 3, 280–285.

[pala12615-bib-0086] Jiang, T. , Wan, X. , Aitchison, J. C. , Xi, D. and Cao, W. 2018. Foraminiferal response to the PETM recorded in the SW Tarim Basin, central Asia. Palaeogeography, Palaeoclimatology, Palaeoecology, 506, 217–225.

[pala12615-bib-0087] Kamikuri, S. and Wade, B. S. 2012. Radiolarian magnetobiochronology and faunal turnover across the middle/late Eocene boundary at Ocean Drilling Program Site 1052 in the western North Atlantic Ocean. Marine Micropaleontology, 88–89, 41–53.

[pala12615-bib-0088] Kass, R. E. and Raftery, A. E. 1995. Bayes factors. Journal of the American Statistical Association, 90, 773–795.

[pala12615-bib-0089] Kelly, D. C. , Bralower, T. J. and Zachos, J. C. 1998. Evolutionary consequences of the latest Paleocene thermal maximum for tropical planktonic Foraminifera. Palaeogeography, Palaeoclimatology, Palaeoecology, 141, 139–161.

[pala12615-bib-0090] Kennett, J. P. and Stott, L. D. 1991. Abrupt deep‐sea warming, palaeoceanographic changes and benthic extinctions at the end of the Palaeocene. Nature, 353, 225–229.

[pala12615-bib-0091] Kidder, D. L. and Tomescu, I. 2016. Biogenic chert and the Ordovician silica cycle. Palaeogeography, Palaeoclimatology, Palaeoecology, 458, 29–38.

[pala12615-bib-0092] Kling, S. A. and Boltovskoy, D. 1999. Radiolaria phaeodaria. South Atlantic Zooplankton, 1, 231–264.

[pala12615-bib-0093] Kocsis, Á. T. , Kiessling, W. and Pálfy, J. 2014. Radiolarian biodiversity dynamics through the Triassic and Jurassic: implications for proximate causes of the end‐Triassic mass extinction. Paleobiology, 40, 625–639.

[pala12615-bib-0094] Kring, D. A. 2007. The Chicxulub impact event and its environmental consequences at the Cretaceous–Tertiary boundary. Palaeogeography, Palaeoclimatology, Palaeoecology, 255, 4–21.

[pala12615-bib-0095] Kuroda, J. , Ogawa, N. O. , Tanimizu, M. , Coffin, M. F. , Tokuyama, H. , Kitazato, H. and Ohkouchi, N. 2007. Contemporaneous massive subaerial volcanism and Late Cretaceous Oceanic Anoxic Event 2. Earth & Planetary Science Letters, 256, 211–223.

[pala12615-bib-0096] Lang, D. T. 2020. RCurl: general network (HTTP/FTP/…) client interface for R. R package v1.98‐1.2. https://cran.r‐project.org/package=RCurl

[pala12615-bib-0097] Larson, R. L. and Erba, E. 1999. Onset of the Mid‐Cretaceous greenhouse in the Barremian‐Aptian: igneous events and the biological, sedimentary, and geochemical responses. Paleoceanography, 14, 663–678.

[pala12615-bib-0098] Lazarus, D. , Barron, J. , Renaudie, J. , Diver, P. and Türke, A. 2014. Cenozoic planktonic marine diatom diversity and correlation to climate change. PLoS One, 9, e84857. 24465441 10.1371/journal.pone.0084857 PMC3898954

[pala12615-bib-0099] Leckie, R. M. 1989. A paleoceanographic model for the early evolutionary history of planktonic Foraminifera. Palaeogeography, Palaeoclimatology, Palaeoecology, 73, 107–138.

[pala12615-bib-0100] Leckie, R. M. , Bralower, T. J. and Cashman, R. 2002. Oceanic anoxic events and plankton evolution: biotic response to tectonic forcing during the mid‐Cretaceous. Paleoceanography, 17, 13‐1–13–29.

[pala12615-bib-0101] Li, Y.‐X. , Bralower, T. J. , Montañez, I. P. , Osleger, D. A. , Arthur, M. A. , Bice, D. M. , Herbert, T. D. , Erba, E. and Premoli Silva, I. 2008. Toward an orbital chronology for the early Aptian Oceanic Anoxic Event (OAE1a, ~120 Ma). Earth & Planetary Science Letters, 271, 88–100.

[pala12615-bib-0102] Lini, A. , Weissert, H. and Erba, E. 1992. The Valanginian carbon isotope event: a first episode of greenhouse climate conditions during the Cretaceous. Terra Nova, 4, 374–384.

[pala12615-bib-0103] Liow, H. and Stenseth, N. C. 2007. The rise and fall of species: implications for macroevolutionary and macroecological studies. Proceedings of the Royal Society B, 274, 2745–2752. 17711843 10.1098/rspb.2007.1006 PMC2279224

[pala12615-bib-0104] Liow, L. H. , Skaug, H. J. , Ergon, T. and Schweder, T. 2010. Global occurrence trajectories of microfossils: environmental volatility and the rise and fall of individual species. Paleobiology, 36, 224–252.

[pala12615-bib-0105] Liu, J. , Aitchison, J. C. and Ali, J. R. 2011. Upper Paleocene radiolarians from DSDP Sites 549 and 550, Goban Spur, NE Atlantic. Palaeoworld, 20, 218–231.

[pala12615-bib-0106] Livermore, R. , Hillenbrand, C. , Meredith, M. and Eagles, G. 2007. Drake Passage and Cenozoic climate: an open and shut case? Geochemistry, Geophysics, Geosystems, 8, Q01005.

[pala12615-bib-0107] Lloyd, G. T. , Young, J. R. and Smith, A. B. 2012a. Comparative quality and fidelity of deep‐sea and land‐based nannofossil records. Geology, 40, 155–158.

[pala12615-bib-0108] Lloyd, G. T. , Pearson, P. N. , Young, J. R. and Smith, A. B. 2012b. Sampling bias and the fossil record of planktonic Foraminifera on land and in the deep sea. Paleobiology, 38, 569–584.

[pala12615-bib-0109] Lowery, C. M. and Fraass, A. J. 2019. Morphospace expansion paces taxonomic diversification after end Cretaceous mass extinction. Nature Ecology & Evolution, 3, 900–904. 30962557 10.1038/s41559-019-0835-0

[pala12615-bib-0110] Lowery, C. M. , Bralower, T. J. , Owens, J. D. , Rodríguez‐Tovar, F. J. , Jones, H. , Smit, J. , Whalen, M. T. , Claeys, P. , Farley, K. , Gulick, S. P. S. , Morgan, J. V. , Green, S. , Chenot, E. , Christeson, G. L. , Cockell, C. S. , Coolen, M. J. L. , Ferrière, L. , Gebhardt, C. , Goto, K. , Kring, D. A. , Lofi, J. , Ocampo‐Torres, R. , Perez‐Cruz, L. , Pickersgill, A. E. , Poelchau, M. H. , Rae, A. S. P. , Rasmussen, C. , Rebolledo‐Vieyra, M. , Riller, U. , Sato, H. , Tikoo, S. M. , Tomioka, N. , Urrutia‐Fucugauchi, J. , Vellekoop, J. , Wittmann, A. , Xiao, L. , Yamaguchi, K. E. and Zylberman, W. 2018. Rapid recovery of life at ground zero of the end‐Cretaceous mass extinction. Nature, 558, 288–291. 29849143 10.1038/s41586-018-0163-6 PMC6058194

[pala12615-bib-0111] Lowery, C. M. , Bown, P. R. , Fraass, A. J. and Hull, P. M. 2020. Ecological response of plankton to environmental change: thresholds for extinction. Annual Review of Earth & Planetary Sciences, 48, 403–429.

[pala12615-bib-0112] Lyle, M. 2003. Neogene carbonate burial in the Pacific Ocean. Paleoceanography, 18, 1059.

[pala12615-bib-0113] Lyle, M. , Lyle, A. O. , Backman, J. and Tripati, A. 2005. Biogenic sedimentation in the Eocene equatorial Pacific—The stuttering greenhouse and Eocene carbonate compensation depth. Proceedings of the Ocean Drilling Program, Scientific Results, 199, 1–35.

[pala12615-bib-0114] Magee, A. F. and Höhna, S. 2021. Impact of K‐Pg mass extinction event on crocodylomorpha inferred from phylogeny of extinct and extant taxa. BioRxiv, 2021.01.14.426715.

[pala12615-bib-0115] Mattioli, E. , Pittet, B. , Riquier, L. and Grossi, V. 2014. The mid‐Valanginian Weissert Event as recorded by calcareous nannoplankton in the Vocontian Basin. Palaeogeography, Palaeoclimatology, Palaeoecology, 414, 472–485.

[pala12615-bib-0116] McInerney, F. A. and Wing, S. L. 2011. The Paleocene‐Eocene thermal maximum: a perturbation of carbon cycle, climate, and biosphere with implications for the future. Annual Review of Earth & Planetary Sciences, 39, 489–516.

[pala12615-bib-0117] Meyers, P. A. , Bernasconi, S. M. and Forster, A. 2006. Origins and accumulation of organic matter in expanded Albian to Santonian black shale sequences on the Demerara Rise, South American margin. Organic Geochemistry, 37, 1816–1830.

[pala12615-bib-0118] Midtkandal, I. , Svensen, H. H. , Planke, S. , Corfu, F. , Polteau, S. , Torsvik, T. H. , Faleide, J. I. , Grundvåg, S.‐A. , Selnes, H. , Kürschner, W. and Olaussen, S. 2016. The Aptian (Early Cretaceous) oceanic anoxic event (OAE1a) in Svalbard, Barents Sea, and the absolute age of the Barremian‐Aptian boundary. Palaeogeography, Palaeoclimatology, Palaeoecology, 463, 126–135.

[pala12615-bib-0119] Mitchell, J. S. , Etienne, R. S. and Rabosky, D. L. 2019. Inferring diversification rate variation from phylogenies with fossils. Systematic Biology, 68, 1–18. 29788398 10.1093/sysbio/syy035

[pala12615-bib-0120] Mitlehner, A. G. 1996. Palaeoenvironments in the North Sea Basin around the Paleocene‐Eocene boundary: evidence from diatoms and other siliceous microfossils. Geological Society, London, Special Publications, 101, 255–273.

[pala12615-bib-0121] Montoya‐Pino, C. , Weyer, S. , Anbar, A. D. , Pross, J. , Oschmann, W. , van de Schootbrugge, B. and Arz, H. W. 2010. Global enhancement of ocean anoxia during Oceanic Anoxic Event 2: a quantitative approach using U isotopes. Geology, 38, 315–318.

[pala12615-bib-0122] Musavu‐Moussavou, B. , Danelian, T. , Baudin, F. , Coccioni, R. and Fröhlich, F. 2007. The Radiolarian biotic response during OAE2. A high‐resolution study across the Bonarelli level at Bottaccione (Gubbio, Italy). Revue de Micropaléontologie, 50, 253–287.

[pala12615-bib-0123] Mutterlose, J. , Linnert, C. and Norris, R. 2007. Calcareous nannofossils from the Paleocene–Eocene Thermal Maximum of the equatorial Atlantic (ODP Site 1260B): evidence for tropical warming. Marine Micropaleontology, 65, 13–31.

[pala12615-bib-0124] Naish, T. R. , Woolfe, K. J. , Barrett, P. J. , Wilson, G. S. , Atkins, C. , Bohaty, S. M. , Bücker, C. J. , Claps, M. , Davey, F. J. , Dunbar, G. B. , Dunn, A. G. , Fielding, C. R. , Florindo, F. , Hannah, M. J. , Harwood, D. M. , Henrys, S. A. , Krissek, L. A. , Lavelle, M. , van der Meer, J. , McIntosh, W. C. , Niessen, F. , Passchier, S. , Powell, R. D. , Roberts, A. P. , Sagnotti, L. , Scherer, R. P. , Strong, C. P. , Talarico, F. , Verosub, K. L. , Villa, G. , Watkins, D. K. , Webb, P.‐N. and Wonik, T. 2001. Orbitally induced oscillations in the East Antarctic ice sheet at the Oligocene/Miocene boundary. Nature, 413, 719–723. 11607028 10.1038/35099534

[pala12615-bib-0125] Norris, R. D. 1996. Symbiosis as an evolutionary innovation in the radiation of Paleocene planktic Foraminifera. Paleobiology, 22, 461–480.

[pala12615-bib-0126] Norris, R. D. 2000. Pelagic species diversity, biogeography, and evolution. Paleobiology, 26, 236–258.

[pala12615-bib-0127] O’Dogherty, L. and Guex, J. 2002. Rates and pattern of evolution among Cretaceous radiolarians: relations with global paleoceanographic events. Micropaleontology, 1–22.

[pala12615-bib-0128] Oreshkina, T. V. 2012. Evidence of late Paleocene–early Eocene hyperthermal events in biosiliceous sediments of Western Siberia and adjacent areas. Australian Journal of Earth Sciences, 105, 145–153.

[pala12615-bib-0129] Oreshkina, T. V. and Radionova, E. P. 2014. Diatom record of the Paleocene–Eocene Thermal Maximum in marine paleobasins of Central Russia, Transuralia and adjacent regions. Nova Hedwigia, Beiheft 143, 307–336.

[pala12615-bib-0130] Pälike, H. , Lyle, M. W. , Nishi, H. , Raffi, I. , Ridgwell, A. , Gamage, K. , Klaus, A. , Acton, G. , Anderson, L. , Backman, J. , Baldauf, J. , Beltran, C. , Bohaty, S. M. , Bown, P. , Busch, W. , Channell, J. E. T. , Chun, C. O. J. , Delaney, M. , Dewangan, P. , Dunkley Jones, T. , Edgar, K. M. , Evans, H. , Fitch, P. , Foster, G. L. , Gussone, N. , Hasegawa, H. , Hathorne, E. C. , Hayashi, H. , Herrle, J. O. , Holbourn, A. , Hovan, S. , Hyeong, K. , Iijima, K. , Ito, T. , Kamikuri, S. I. , Kimoto, K. , Kuroda, J. , Leon‐Rodriguez, L. , Malinverno, A. , Moore, T. C. , Murphy, B. H. , Murphy, D. P. , Nakamura, H. , Ogane, K. , Ohneiser, C. , Richter, C. , Robinson, R. , Rohling, E. J. , Romero, O. , Sawada, K. , Scher, H. , Schneider, L. , Sluijs, A. , Takata, H. , Tian, J. , Tsujimoto, A. , Wade, B. S. , Westerhold, T. , Wilkens, R. , Williams, T. , Wilson, P. A. , Yamamoto, Y. , Yamamoto, S. , Yamazaki, T. and Zeebe, R. E. 2012. A Cenozoic record of the equatorial Pacific carbonate compensation depth. Nature, 488, 609–614. 22932385 10.1038/nature11360

[pala12615-bib-0131] Parente, M. , Frijia, G. , di Lucia, M. , Jenkyns, H. C. , Woodfine, R. G. and Baroncini, F. 2008. Stepwise extinction of larger foraminifers at the Cenomanian‐Turonian boundary: a shallow‐water perspective on nutrient fluctuations during Oceanic Anoxic Event 2 (Bonarelli Event). Geology, 36, 715–718.

[pala12615-bib-0132] Pearson, P. N. , McMillan, I. K. , Wade, B. S. , Jones, T. D. , Coxall, H. K. , Bown, P. R. and Lear, C. H. 2008. Extinction and environmental change across the Eocene‐Oligocene boundary in Tanzania. Geology, 36, 179–182.

[pala12615-bib-0133] Penman, D. E. , Hönisch, B. , Zeebe, R. E. , Thomas, E. and Zachos, J. C. 2014. Rapid and sustained surface ocean acidification during the Paleocene‐Eocene Thermal Maximum. Paleoceanography, 29, 357–369.

[pala12615-bib-0134] Peters, S. E. , Kelly, D. C. and Fraass, A. J. 2013. Oceanographic controls on the diversity and extinction of planktonic Foraminifera. Nature, 493, 398–401. 23302802 10.1038/nature11815

[pala12615-bib-0135] Petrizzo, M. R. 2007. The onset of the Paleocene–Eocene Thermal Maximum (PETM) at Sites 1209 and 1210 (Shatsky Rise, Pacific Ocean) as recorded by planktonic foraminifera. Marine Micropaleontology, 63, 187–200.

[pala12615-bib-0136] Petrizzo, M. R. , Huber, B. T. , Wilson, P. A. and MacLeod, K. G. 2008. Late Albian paleoceanography of the western subtropical North Atlantic. Paleoceanography, 23, PA1517.

[pala12615-bib-0137] Petrizzo, M. R. , Wade, B. and Gradstein, F. 2020. Planktonic Foraminifera. 74–87. *In* Gradstein, F. , Ogg, J. G. , Schmitz, M. and Ogg, G. (eds). Geologic time scale 2020. Elsevier.

[pala12615-bib-0138] Pogge von Strandmann, P. A. E. , Jenkyns, H. C. and Woodfine, R. G. 2013. Lithium isotope evidence for enhanced weathering during Oceanic Anoxic Event 2. Nature Geoscience, 6, 668–672.

[pala12615-bib-0139] Premoli Silva, I. and Sliter, W. V. 1999. Cretaceous paleoceanography: evidence from planktonic foraminiferal evolution. 301–328. *In* Barrera, E. and Johnson, C. C. (eds). Evolution of the Cretaceous ocean‐climate system. Geological Society of America.

[pala12615-bib-0140] R Core Team 2013. R: a language and environment for statistical computing. R Foundation for Statistical Computing. https://www.R‐project.org

[pala12615-bib-0141] Rabosky, D. L. and Sorhannus, U. 2009. Diversity dynamics of marine planktonic diatoms across the Cenozoic. Nature, 457, 183–186. 19129846 10.1038/nature07435

[pala12615-bib-0142] Raffi, I. , Backman, J. and Pälike, H. 2005. Changes in calcareous nannofossil assemblages across the Paleocene/Eocene transition from the paleo‐equatorial Pacific Ocean. Palaeogeography, Palaeoclimatology, Palaeoecology, 226, 93–126.

[pala12615-bib-0143] Rambaut, A. , Drummond, A. J. , Xie, D. , Baele, G. and Suchard, M. A. 2018. Posterior summarization in Bayesian phylogenetics using Tracer 1.7. Systematic Biology, 67, 901. 29718447 10.1093/sysbio/syy032 PMC6101584

[pala12615-bib-0144] Raup, D. M. and Sepkoski, J. J. 1982. Mass extinctions in the marine fossil. Science, 215, 1501–1503. 17788674 10.1126/science.215.4539.1501

[pala12615-bib-0145] Renaudie, J. and Lazarus, D. B. 2013. On the accuracy of paleodiversity reconstructions: a case study in Antarctic Neogene radiolarians. Paleobiology, 39, 491–509.

[pala12615-bib-0146] Renaudie, J. , Lazarus, D. and Diver, P. 2020. NSB (Neptune Sandbox Berlin): an expanded and improved database of marine planktonic microfossil data and deep‐sea stratigraphy. Palaeontologia Electronica, 23 (1), a11.

[pala12615-bib-0147] Ridgwell, A. and Schmidt, D. N. 2010. Past constraints on the vulnerability of marine calcifiers to massive carbon dioxide release. Nature Geoscience, 3, 196–200.

[pala12615-bib-0148] Ridgwell, A. and Zeebe, R. E. 2005. The role of the global carbonate cycle in the regulation and evolution of the Earth system. Earth & Planetary Science Letters, 234, 299–315.

[pala12615-bib-0149] Rivero‐Cuesta, L. , Westerhold, T. , Agnini, C. , Dallanave, E. , Wilkens, R. H. and Alegret, L. 2019. Paleoenvironmental changes at ODP Site 702 (South Atlantic): anatomy of the Middle Eocene Climatic Optimum. Paleoceanography & Paleoclimatology, 34, 2047–2066.

[pala12615-bib-0150] Robinson, S. A. , Williams, T. and Bown, P. R. 2004. Fluctuations in biosiliceous production and the generation of Early Cretaceous oceanic anoxic events in the Pacific Ocean (Shatsky Rise, Ocean Drilling Program Leg 198). Paleoceanography, 19, PA4024.

[pala12615-bib-0151] Rodríguez‐Cuicas, M.‐E. , Montero‐Serrano, J.‐C. and Garbán, G. 2019. Paleoenvironmental changes during the late Albian oceanic anoxic event 1d: an example from the Capacho Formation, southwestern Venezuela. Palaeogeography, Palaeoclimatology, Palaeoecology, 521, 10–29.

[pala12615-bib-0152] Scheibner, C. , Speijer, R. P. and Marzouk, A. M. 2005. Turnover of larger Foraminifera during the Paleocene‐Eocene Thermal Maximum and paleoclimatic control on the evolution of platform ecosystems. Geology, 33, 493–496.

[pala12615-bib-0153] Schueth, J. D. , Bralower, T. J. , Jiang, S. and Patzkowsky, M. E. 2015. The role of regional survivor incumbency in the evolutionary recovery of calcareous nannoplankton from the Cretaceous/Paleogene (K/Pg) mass extinction. Paleobiology, 41, 661–679.

[pala12615-bib-0154] Schulte, P. , Alegret, L. , Arenillas, I. , Arz, J. A. , Barton, P. J. , Bown, P. R. , Bralower, T. J. , Christeson, G. L. , Claeys, P. and Cockell, C. S. 2010. The Chicxulub asteroid impact and mass extinction at the Cretaceous‐Paleogene boundary. Science, 327, 1214–1218. 20203042 10.1126/science.1177265

[pala12615-bib-0155] Shevenell, A. E. , Kennett, J. P. and Lea, D. W. 2004. Middle Miocene southern ocean cooling and antarctic cryosphere expansion. Science, 305, 1766–1770. 15375266 10.1126/science.1100061

[pala12615-bib-0156] Silvestro, D. , Schnitzler, J. , Liow, L. H. , Antonelli, A. and Salamin, N. 2014a. Bayesian estimation of speciation and extinction from incomplete fossil occurrence data. Systematic Biology, 63, 349–367. 24510972 10.1093/sysbio/syu006 PMC4361715

[pala12615-bib-0157] Silvestro, D. , Salamin, N. and Schnitzler, J. 2014b. PyRate: a new program to estimate speciation and extinction rates from incomplete fossil data. Methods in Ecology & Evolution, 5, 1126–1131.

[pala12615-bib-0158] Silvestro, D. , Warnock, R. C. M. , Gavryushkina, A. and Stadler, T. 2018. Closing the gap between palaeontological and neontological speciation and extinction rate estimates. Nature Communications, 9, 5237. 10.1038/s41467-018-07622-y PMC6286320 30532040

[pala12615-bib-0159] Silvestro, D. , Salamin, N. , Antonelli, A. and Meyer, X. 2019. Improved estimation of macroevolutionary rates from fossil data using a Bayesian framework. Paleobiology, 45, 1–25.

[pala12615-bib-0160] Smart, C. W. and Thomas, E. 2006. The enigma of early Miocene biserial planktic foraminifera. Geology, 34, 1041–1044.

[pala12615-bib-0161] Soua, M. 2016. Cretaceous oceanic anoxic events (OAEs) recorded in the northern margin of Africa as possible oil and gas shale potential in Tunisia: an overview. International Geology Review, 58, 277–320.

[pala12615-bib-0162] Spencer‐Cervato, C. 1999. The Cenozoic deep sea microfossil record: explorations of the DSDP/ODP sample set using the Neptune database. Palaeontologia Electronica, 2, 270.

[pala12615-bib-0163] Spencer‐Cervato, C. , Thierstein, H. R. , Lazarus, D. B. and Beckmann, J. 1994. How synchronous are Neogene marine plankton events? Paleoceanography, 9, 739–763.

[pala12615-bib-0164] Stadler, T. , Gavryushkina, A. , Warnock, R. C. M. , Drummond, A. J. and Heath, T. A. 2018. The fossilized birth‐death model for the analysis of stratigraphic range data under different speciation modes. Journal of Theoretical Biology, 447, 41–55. 29550451 10.1016/j.jtbi.2018.03.005 PMC5931795

[pala12615-bib-0165] Stickley, C. E. and Koc, N. 2009. The Big Freeze: Diatoms record Arctic sea ice at 47 Ma. EGU General Assembly Conference Abstracts, 1665.

[pala12615-bib-0166] Stickley, C. E. , St John, K. , Koç, N. , Jordan, R. W. , Passchier, S. , Pearce, R. B. and Kearns, L. E. 2009. Evidence for middle Eocene Arctic sea ice from diatoms and ice‐rafted debris. Nature, 460, 376–379. 19606146 10.1038/nature08163

[pala12615-bib-0167] Strong, C. P. , Hollis, C. J. and Wilson, G. J. 1995. Foraminiferal, radiolarian, and dinoflagellate biostratigraphy of Late Cretaceous to Middle Eocene pelagic sediments (Muzzle Group), Mead Stream, Marlborough, New Zealand. New Zealand Journal of Geology & Geophysics, 38, 171–209.

[pala12615-bib-0168] Suto, I. 2006. The explosive diversification of the diatom genus *Chaetoceros* across the Eocene/Oligocene and Oligocene/Miocene boundaries in the Norwegian Sea. Marine Micropaleontology, 58, 259–269.

[pala12615-bib-0169] Tabor, C. R. , Bardeen, C. G. , Otto‐Bliesner, B. L. , Garcia, R. R. and Toon, O. B. 2020. Causes and climatic consequences of the impact winter at the Cretaceous‐Paleogene boundary. Geophysical Research Letters, 47, e60121.

[pala12615-bib-0170] Tiraboschi, D. , Erba, E. and Jenkyns, H. C. 2009. Origin of rhythmic Albian black shales (Piobbico core, central Italy): calcareous nannofossil quantitative and statistical analyses and paleoceanographic reconstructions. Paleoceanography, 24, PA2222.

[pala12615-bib-0171] Turgeon, S. C. and Creaser, R. A. 2008. Cretaceous oceanic anoxic event 2 triggered by a massive magmatic episode. Nature, 454, 323–326. 18633415 10.1038/nature07076

[pala12615-bib-0172] Vahlenkamp, M. , Niezgodzki, I. , de Vleeschouwer, D. , Bickert, T. , Harper, D. , Kirtland Turner, S. , Lohmann, G. , Sexton, P. , Zachos, J. and Pälike, H. 2018. Astronomically paced changes in deep‐water circulation in the western North Atlantic during the middle Eocene. Earth & Planetary Science Letters, 484, 329–340.

[pala12615-bib-0173] Van Andel, T. H. 1975. Mesozoic/Cenozoic calcite compensation depth and the global distribution of calcareous sediments. Earth & Planetary Science Letters, 26, 187–194.

[pala12615-bib-0174] Van Cappellen, P. and Qiu, L. 1997. Biogenic silica dissolution in sediments of the Southern Ocean. II. Kinetics. Deep Sea Research Part II: Topical Studies in Oceanography, 44, 1129–1149.

[pala12615-bib-0175] Vellekoop, J. , Sluijs, A. , Smit, J. , Schouten, S. , Weijers, J. W. H. , Sinninghe Damsté, J. S. and Brinkhuis, H. 2014. Rapid short‐term cooling following the Chicxulub impact at the Cretaceous–Paleogene boundary. Proceedings of the National Academy of Sciences, 111, 7537–7541. 10.1073/pnas.1319253111 PMC4040585 24821785

[pala12615-bib-0176] Vellekoop, J. , Esmeray‐Senlet, S. , Miller, K. G. , Browning, J. V. , Sluijs, A. , Van De Schootbrugge, B. , Damsté, J. S. S. and Brinkhuis, H. 2016. Evidence for Cretaceous‐Paleogene boundary bolide “impact winter” conditions from New Jersey, USA. Geology, 44, 619–622.

[pala12615-bib-0177] Wade, B. S. and Kroon, D. 2002. Middle Eocene regional climate instability: evidence from the western North Atlantic. Geology, 30, 1011–1014.

[pala12615-bib-0178] Wade, B. S. and Pearson, P. N. 2008. Planktonic foraminiferal turnover, diversity fluctuations and geochemical signals across the Eocene/Oligocene boundary in Tanzania. Marine Micropaleontology, 68, 244–255.

[pala12615-bib-0179] Wang, T. , Li, G. , Aitchison, J. C. , Ding, L. and Sheng, J. 2019. Evolution of mid‐Cretaceous radiolarians in response to oceanic anoxic events in the eastern Tethys (southern Tibet, China). Palaeogeography, Palaeoclimatology, Palaeoecology, 536, 109369.

[pala12615-bib-0180] Warnock, R. C. M. , Parham, J. F. , Joyce, W. G. , Lyson, T. R. and Donoghue, P. C. J. 2015. Calibration uncertainty in molecular dating analyses: there is no substitute for the prior evaluation of time priors. Proceedings of the Royal Society B, 282, 20141013. 25429012 10.1098/rspb.2014.1013 PMC4262156

[pala12615-bib-0181] Watkins, D. K. , Cooper, M. J. and Wilson, P. A. 2005. Calcareous nannoplankton response to late Albian oceanic anoxic event 1d in the western North Atlantic. Paleoceanography, 20, PA2010.

[pala12615-bib-0182] Weissert, H. 1989. C‐Isotope stratigraphy, a monitor of paleoenvironmental change: a case study from the early cretaceous. Surveys in Geophysics, 10, 1–61.

[pala12615-bib-0183] Wickham, H. , Averick, M. , Bryan, J. , Chang, W. , McGowan, L. , Francois, R. , Grolemund, G. , Hayes, A. , Henry, L. , Hester, J. , Kuhn, M. , Pederson, T. , Miller, E. , Bache, S. , Muller, K. , Ooms, J. , Robinson, D. , Seidel, D. , Spinu, V. , Takahashi, K. , Vaughan, D. , Wilke, C. , Woo, K. and Yutani, H. 2019. Welcome to the tidyverse. Journal of Open Source Software, 4, 1686.

[pala12615-bib-0184] Wickham, H. , Hester, J. , Chang, W. and Hester, M. J. 2020. devtools: tools to make developing R packages easier. R package v2.4.2. https://cran.r‐project.org/package=devtools

[pala12615-bib-0185] Williams, J. R. and Bralower, T. J. 1995. Nannofossil assemblages, fine fraction stable isotopes, and the paleoceanography of the Valanginian‐Barremian (early Cretaceous) North Sea Basin. Paleoceanography, 10, 815–839.

[pala12615-bib-0186] Wilson, P. A. and Norris, R. D. 2001. Warm tropical ocean surface and global anoxia during the mid‐Cretaceous period. Nature, 412, 425–429. 11473314 10.1038/35086553

[pala12615-bib-0187] Witkowski, J. , Bohaty, S. M. , Edgar, K. M. and Harwood, D. M. 2014. Rapid fluctuations in mid‐latitude siliceous plankton production during the Middle Eocene Climatic Optimum (ODP Site 1051, western North Atlantic). Marine Micropaleontology, 106, 110–129.

[pala12615-bib-0188] Wolbach, W. S. , Gilmour, I. and Anders, E. 1990. Major wildfires at the Cretaceous/Tertiary boundary. Geological Society of America Special Paper, 247, 391–400.

[pala12615-bib-0189] Wortmann, U. G. and Weissert, H. 2000. Tying platform drowning to perturbations of the global carbon cycle with a δ13COrg‐curve from the Valanginian of DSDP Site 416. Terra Nova, 12, 289–294.

[pala12615-bib-0190] Yapp, C. J. 2004. Fe(CO_3_)OH in goethite from a mid‐latitude North American Oxisol: estimate of atmospheric CO_2_ concentration in the Early Eocene “climatic optimum”. Geochimica et Cosmochimica Acta, 68, 935–947.

[pala12615-bib-0191] Zachos, J. C. , Pagani, M. , Sloan, L. , Thomas, E. and Billups, K. 2001a. Trends, rhythms, and aberrations in global climate 65 Ma to present. Science, 292, 686–693. 11326091 10.1126/science.1059412

[pala12615-bib-0192] Zachos, J. C. , Shackleton, N. J. , Revenaugh, J. S. , Pälike, H. and Flower, B. P. 2001b. Climate response to orbital forcing across the Oligocene‐Miocene boundary. Science, 292, 274–278. 11303100 10.1126/science.1058288

[pala12615-bib-0193] Zachos, J. C. , Dickens, G. R. and Zeebe, R. E. 2008. An early Cenozoic perspective on greenhouse warming and carbon‐cycle dynamics. Nature, 451, 279–283. 18202643 10.1038/nature06588

[pala12615-bib-0194] Zeebe, R. E. and Zachos, J. C. 2013. Long‐term legacy of massive carbon input to the Earth system: anthropocene versus Eocene. Philosophical Transactions of the Royal Society A, 371, 20120006. 10.1098/rsta.2012.0006 24043863

[pala12615-bib-0195] Zou, Z. , Huang, C. , Li, M. and Zhang, Y. 2016. Climate change response to astronomical forcing during the Oligocene‐Miocene transition in the equatorial Atlantic (ODP Site 926). Science China Earth Sciences, 59, 1665–1673.

