# Supplementary figures and images for: Diversity dynamics of microfossils from the Cretaceous to the Neogene show mixed responses to events
Source: Palaeontology. 2022 Jul 15;65(4):e12615. doi: 10.1111/pala.12615 (PMC9540813; doi:10.1111/pala.12615)

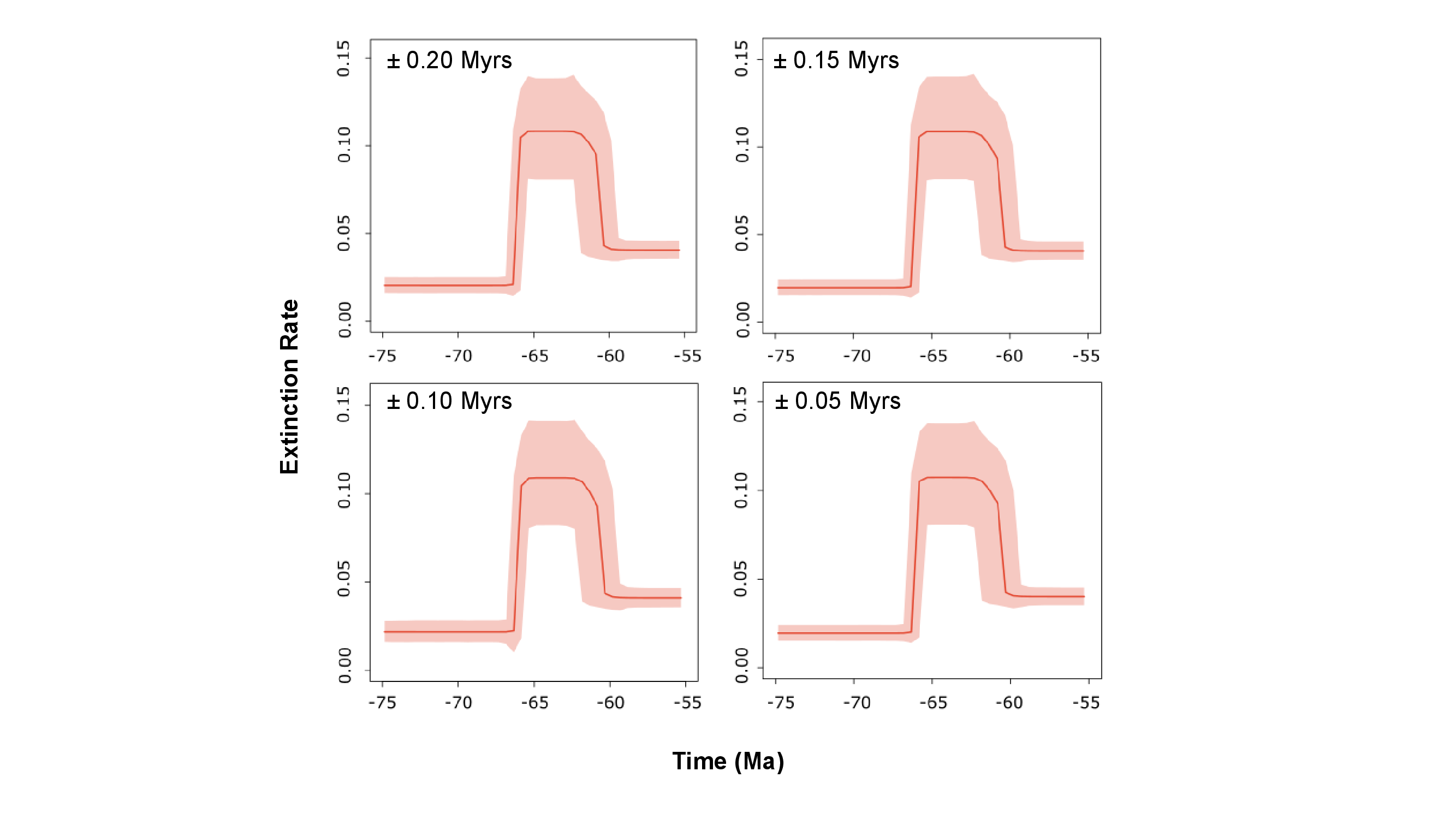

Supplement: Supplementary file 1 — Fig. S1. Sensitivity of PyRate analyses to occurrence age. [file PALA-65-0-s005.tiff]

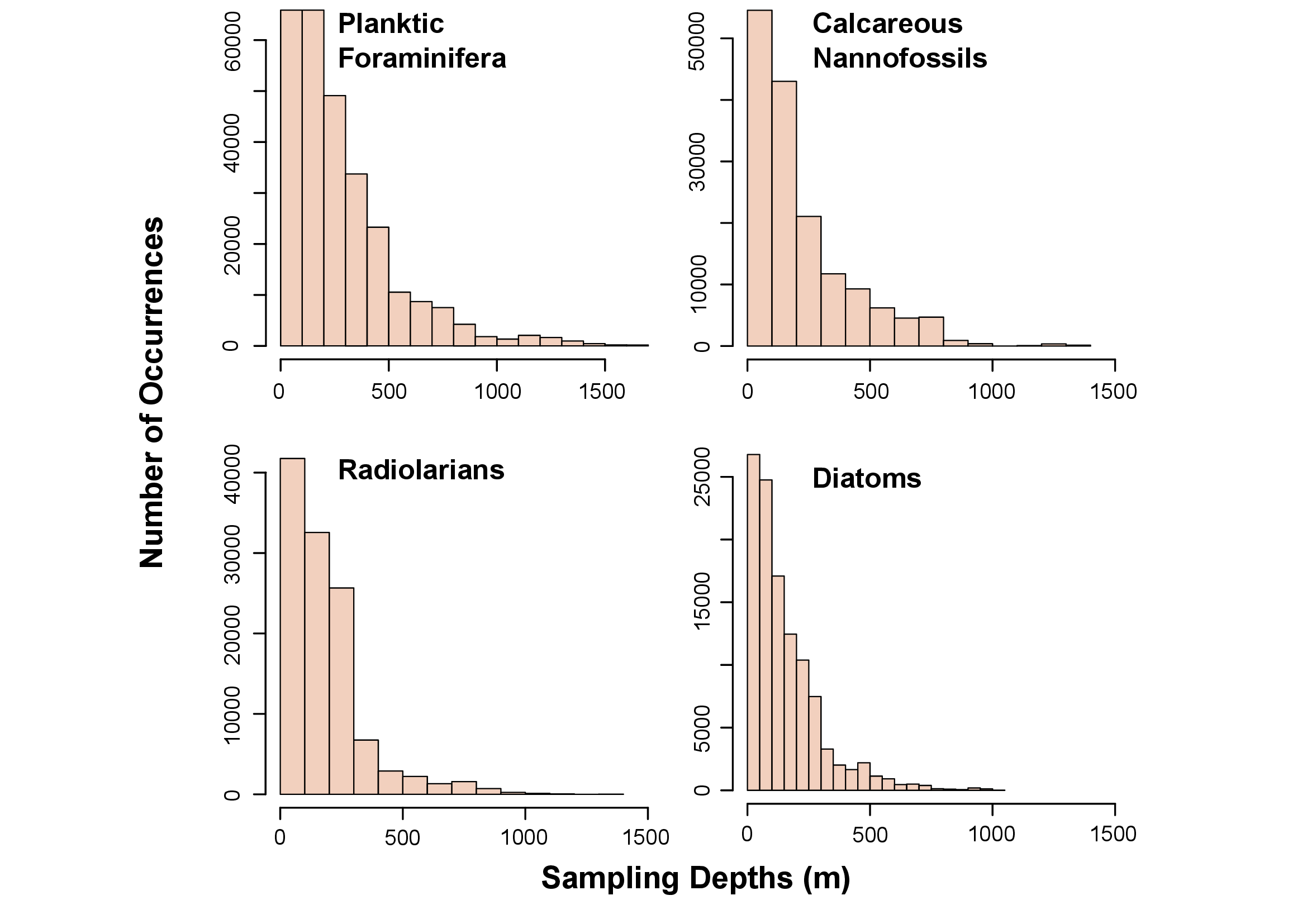

Supplement: Supplementary file 2 — Fig. S2. Number of species occurrences at different sampling depths. [file PALA-65-0-s002.tiff]

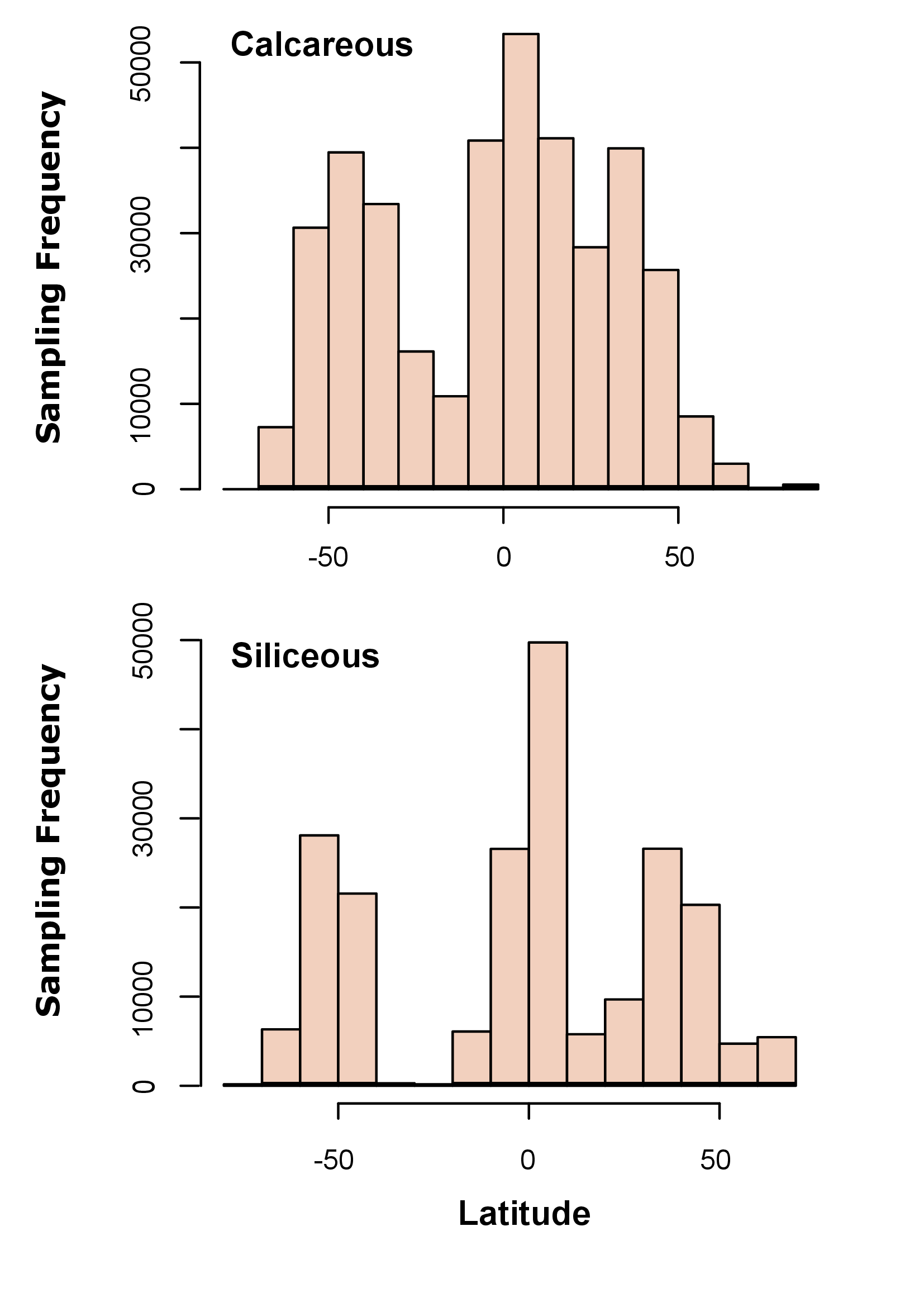

Supplement: Supplementary file 3 — Fig. S3. Latitudinal differences in sampling frequency of microfossil occurrences. [file PALA-65-0-s004.tiff]
