# Supplementary material for: Diversity dynamics of microfossils from the Cretaceous to the Neogene show mixed responses to events
Source: Palaeontology. 2022 Jul 15;65(4):e12615. doi: 10.1111/pala.12615 (PMC9540813; doi:10.1111/pala.12615)
Supplement: Supplementary file 4 — Data S1. Example data extraction from Neptune Sandbox Berlin. [file PALA-65-0-s001.docx]

**Example Data Extraction from NSB**

#Data Collection

#Retrieving packages needed from library

library(data.table)

library(devtools)

library(RCurl)

library(tidyverse)

#Set Working Directory

setwd("C:/Users/User_Name/Documents/Nannofossil Collection")

#NSB data access

library(NSBcompanion)

#Connecting account to the network

nsb <-nsbConnect("username", "password”, intern=FALSE, local=FALSE)

#Retrieving nannos data

NannosAll <- getNeptuneData(nsb, fossil_group = "N")

#Resolving synonymy

NannosSynon <- NSBcompanion::resolveSynonymy(nsb, NannosAll)

#Removing non-valid taxa

which(NannosSynon$resolved_taxon_status == "V")

VALIDNannos <-NannosSynon[which(NannosSynon$resolved_taxon_status == "V"), ]

#Write CSV

write.csv(VALIDNannos, file = "NannosAll.csv", row.names = FALSE)

#Converting format suitable for PyRate and adding error margins

dat <- read.csv("NannosAll.csv", header = TRUE) %>%

filter(taxon_status != "S")

new_dat <- dat %>%

transmute(

taxon_name = paste0(genus, "_", species),

status = rep("extinct", nrow(dat)),

MinAge = sample_age_ma - 0.25, MaxAge = sample_age_ma + 0.25,

fossil_group

)

new_dat[which(new_dat$MinAge < 0), c("MinAge", "MaxAge")] <- 0

head(new_dat)

write.table(new_dat, "NannosFormatted.tsv", sep = "\t", row.names = FALSE)

write.csv(new_dat, file = "NannosFormatted.csv", row.names = FALSE)

```

#Removing NAs in the dataset

NANNOSALL_NA <- na.omit(NannosAllFinal[c(1,2,3,4)])

write.csv(NANNOSALL_NA, file ="NANNOS_NA.csv", row.names = FALSE)

NANNOS_NA <- na.omit(Nannos25[c(1,2,3,4)])

#Establishing which taxa are extant and those that are extinct

extant_tax_nannos <- NANNOS_NA %>%

filter(MinAge == 0)

extant_rows_nannos <- which(NANNOS_NA$taxon_name %in% extant_tax_nannos$taxon_name)

NANNOS_NA$status <- rep("extinct", )

NANNOS_NA$status[extant_rows_nannos] <- "extant"

#Write updated table and CSV to input into PyRate

write.table(NANNOSALL_NA, "ALLNANNOSFINAL.tsv", sep = "\t", row.names = FALSE)

write.csv(NANNOSALL_NA, "ALLNANNOSFINAL.csv", row.names = FALSE)
