# Supplementary material for: Diversity dynamics of microfossils from the Cretaceous to the Neogene show mixed responses to events
Source: Palaeontology. 2022 Jul 15;65(4):e12615. doi: 10.1111/pala.12615 (PMC9540813; doi:10.1111/pala.12615)
Supplement: Supplementary file 5 — Data S2. Example PyRate Script used for diversification analysis. [file PALA-65-0-s003.docx]

**Example PyRate Script utilised for diversification analysis.**

*The name of the Python file created via pyrate utilities is added to the final line of the script.*

#!/bin/bash

### PyRate forams analysis

### 10 runs in an array job

### name of job

#PBS -N pyrate_Foraminifera_mHPP_mG

### time to stop job

#PBS -l walltime=72:00:00

### number of nodes/cpus to use *for each job*

### in this case each run of PyRate uses 1 CPU

#PBS -l select=1:ncpus=1:mem=8gb

### index range for subjobs

### this is how many runs of PyRate we want

#PBS -J 1-10

module add lang/python/anaconda/3.7.7-2020-R-3.6.1

python3 /work/username/PyRate/PyRate.py /work/username/Foraminifera_PyRate.py -mHPP -mG -n 100000000 -s 5000 -j $PBS_ARRAY_INDEX
